# Patient-Generated Health Photos and Videos Across Health and Well-being Contexts: Scoping Review

**DOI:** 10.2196/28867

**Published:** 2022-04-12

**Authors:** Bernd Ploderer, Atae Rezaei Aghdam, Kara Burns

**Affiliations:** 1 School of Computer Science Queensland University of Technology Brisbane Australia; 2 School of Information Systems Queensland University of Technology Brisbane Australia; 3 Centre for Digital Transformation of Health Faculty of Medicine, Dentistry and Health Sciences The University of Melbourne Melbourne Australia

**Keywords:** patient engagement, patient-generated health data, consumer-generated health data, personal health information, patient empowerment, mobile phone

## Abstract

**Background:**

Patient-generated health data are increasingly used to record health and well-being concerns and engage patients in clinical care. Patient-generated photographs and videos are accessible and meaningful to patients, making them especially relevant during the current COVID-19 pandemic. However, a systematic review of photos and videos used by patients across different areas of health and well-being is lacking.

**Objective:**

This review aims to synthesize the existing literature on the health and well-being contexts in which patient-generated photos and videos are used, the value gained by patients and health professionals, and the challenges experienced.

**Methods:**

Guided by a framework for scoping reviews, we searched eight health databases (CINAHL, Cochrane Library, Embase, PsycINFO, PubMed, MEDLINE, Scopus, and Web of Science) and one computing database (ACM), returning a total of 28,567 studies. After removing duplicates and screening based on the predefined inclusion criteria, we identified 110 relevant articles. Data were charted and articles were analyzed following an iterative thematic approach with the assistance of NVivo software (version 12; QSR International).

**Results:**

Patient-generated photos and videos are used across a wide range of health care services (39/110, 35.5% articles), for example, to diagnose skin lesions, assess dietary intake, and reflect on personal experiences during therapy. In addition, patients use them to self-manage health and well-being concerns (33/110, 30%) and to share personal health experiences via social media (36/110, 32.7%). Photos and videos create significant value for health care (59/110, 53.6%), where images support diagnosis, explanation, and treatment (functional value). They also provide value directly to patients through enhanced self-determination (39/110, 35.4%), social (33/110, 30%), and emotional support (21/110, 19.1%). However, several challenges emerge when patients create, share, and examine photos and videos, such as limited accessibility (16/110, 14.5%), incomplete image sets (23/110, 20.9%), and misinformation through photos and videos shared on social media (17/110, 15.5%).

**Conclusions:**

This review shows that photos and videos engage patients in meaningful ways across different health care activities (eg, diagnosis, treatment, and self-care) for various health conditions. Although photos and videos require effort to capture and involve challenges when patients want to use them in health care, they also engage and empower patients, generating unique value. This review highlights areas for future research and strategies for addressing these challenges.

## Introduction

### Background

There has been a growing interest in patient-generated health data (PGHD) in recent years, where patients create and collect personal information about some aspects of their own health outside the health care setting [1]. This interest has been spurred by technological developments, most notably by sensors embedded in smartphones and wearable devices that allow people to automatically generate a wide range of health data, from physical activity to heart rate to sleep [2-4]. At the same time, patient perspectives are progressively changing from passive recipients of health care to active agents, with an emphasis on proactive well-being, rather than reactive clinical care [5].

Current evidence suggests that for patients, PGHD support the self-management of disease, promote partnership with providers, enable people to gain social support within the peer network, and facilitate the creation of different types of value [6-9]. Health service providers are also increasingly interested in assessing patient health outside the health care setting, for example, through patient-reported outcome measures (PROMs) [10]. In contrast to PROMs, PGHD can be initiated by patients rather than by health care providers. Not only are patients responsible for capturing personal data but they can also direct the sharing of this information and retain ownership of their data [1]. Furthermore, PROMs are often survey-based, whereas PGHD can be diverse, including sensor data, personal diaries, photos, and histories [1,8,11].

This paper focuses on patient-generated photos and videos because they are more accessible and meaningful for patients than other forms of PGHD. First, accessibility stems from the widespread availability of cameras in smartphones, which allows patients to capture photos or videos of their bodies, lifestyles, and experiences relevant for their health and well-being [12]. Photos are also accessible as a medium that patients can readily use and understand across different languages and cultures, without requiring in-depth medical or technical expertise. For example, patients tracking their diet may find it easier to take a photo of each meal consumed than to keep a diary of the ingredients and nutritional value of each meal [13]. In writing that “seeing comes before words,” Berger [14] highlights that photos and videos are accessible on a more fundamental level, because we experience the world, and thereby our health, primarily through our senses, including our visual sense. Second, photos and videos are meaningful for patients because they can communicate something that they cannot directly express, as suggested by Haines et al [15]: “photographs can reveal the gap between ‘what we see and what we know’, and show aspects of experience not easily captured through words alone.” Videos allow patients to discuss and record what they see and experience. Both photos and videos can aid patients in capturing and discussing unique information during consultations, and conversely, they offer prompts to health care professionals to ask questions that may not be asked otherwise. Furthermore, social media (eg, YouTube and PatientsLikeMe) allow patients to share not only data but also personal experiences and knowledge through photos and videos, which make them interesting resources for other patients, health care professionals, and health care organizations [6,16].

During the current COVID-19 global pandemic, the accessibility and meaningfulness of photos and videos are especially relevant. With unprecedented stress on health systems and risk of infection spread, patients and health care providers are looking for tools that are easy to use and accessible for diagnosis and ongoing care via telehealth [17]. However, current systematic reviews on the use of photography and videos for health and well-being concerns have been limited to specific populations [18], a single clinical assessment [13], or one type of content [19]. A comprehensive assessment of the extent of research evidence and the potential scope of patient-generated photos and videos in different areas of health and well-being is lacking.

### Objectives

The overarching objective of this review is to synthesize the literature on patient-generated photos and videos across health and well-being contexts. Specific objectives include (1) providing an overview of the different contexts in which photos and videos are used, (2) examining the value gained for patients and health care professionals, and (3) examining the challenges experienced by these groups in creating, sharing, and examining photos and videos. Throughout the review, we examine the differences between photos and videos. On the basis of these insights, this study seeks to offer practical implications for patients and health care professionals, as well as future research directions for medical informatics researchers.

## Methods

### Overview

This study was guided by the 5-step framework for scoping reviews by Arksey and O’Malley [20]. Scoping reviews aim to comprehensively assess the size and scope of available research literature to convey the breadth of a nascent field. Similar to systematic reviews, scoping reviews aim to be systematic, transparent, and replicable [21]. However, a scoping review protocol has not yet been published. In contrast to systematic reviews, scoping reviews do not assess the quality of included studies because of the paucity of randomized controlled studies [22], and the review also requires analytical reinterpretation of the literature [23]. In the following sections, we describe each of the 5 steps taken to conduct a scoping review of patient-generated photos and videos for health and well-being. For a succinct summary via a scoping review checklist, see Multimedia Appendix 1 [24]. Although the steps are presented in a linear order, it is important to note that the scoping process is iterative and requires a back-and-forth within and between steps as researchers gain a better understanding of the literature [20,22].

### Step 1: Identifying the Research Question

The research questions for this review were as follows: (1) *In which health and well-being contexts are patient-generated photos and videos used?* (2) *What value and challenges do patient-generated photos and videos hold for patients and health care professionals?* These questions were based on our shared interest in photos taken by patients for health, also known as *medical selfies* [12,25]. We refined the questions over time as we became acquainted with the literature to focus on patient-generated photos, rather than selfies, to align with the widespread use of the term *PGHD* in the literature [7,26,27]. Videos were also included because they were similarly captured through smartphones and used in ways similar to photos. Our primary concern has always been with the experiences of patients and their caregivers, as well as their photo-mediated interactions with health care professionals (eg, clinicians, allied health, and nurses) and peers (eg, via social media), rather than a health system or pure technology perspective. On the basis of the literature reviewed, we refined the research question from experience to the more specific study objectives of (1) contexts, (2) value, and (3) challenges.

### Step 2: Identifying Relevant Studies

We devised a systematic search strategy to identify relevant studies. The strategy was based on the literature review of the PhD thesis of the third author (KB) and the support of a librarian. The search terms described in Textbox 1 were based on keywords in the research question and were developed in consultation with a research librarian. Full search strings with particular terms for each database can be found in Multimedia Appendix 2.

**Search terms.**

**Search terms**

(image* OR pictur* OR photo* OR video* OR selfie* OR portrait* OR snap* OR shot* OR depict* OR data* OR info*)

AND

(patient* OR consumer* OR care* OR customer* OR veteran* OR client* OR self* OR crowd*)

AND

(generate* OR record* OR creat* OR captur* OR document* OR evidence* OR story OR report* OR track* OR initiat* OR monitor* OR take*)

The search included articles from January 2008 to January 31, 2021, written in the English language. The start date was chosen because the major brands of smartphones—iPhone (Apple Inc) and Android (Open Handset Alliance)—were first released in 2007 and 2008, respectively, which provide the platform for patient-generated photos and videos. Articles written in other languages were excluded because of the cost and time required for translation. Only peer-reviewed articles that included primary research were selected to ensure that the conclusions were supported by an evidence base.

**Figure 1:**
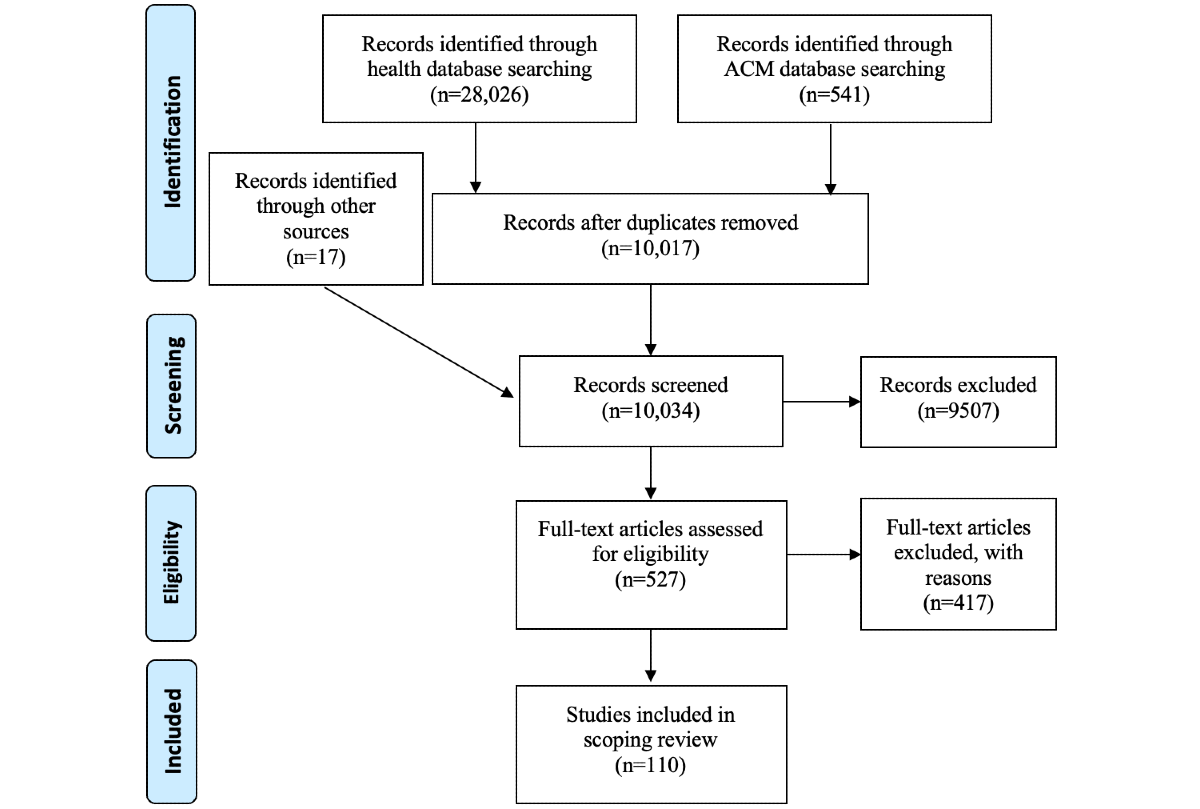
PRISMA (Preferred Reporting Items for Systematic Reviews and Meta-Analyses) flow diagram for the selection of studies from the databases.

According to the objectives of this study and the focus on patients as technology users, we conducted our search strategy in both health and computing databases. Furthermore, we considered social science databases such as Embase and PsycINFO to cover special studies in psychology and behavioral science. We searched eight health databases (CINAHL, Cochrane Library, Embase, PsycINFO, Web of Science, PubMed, MEDLINE, and Scopus) and one computing database (ACM). To ensure that we did not neglect any relevant articles, we broadened the search by using Medical Subject Headings terms and synonyms to collect a comprehensive pool of relevant articles. As illustrated in Figure 1, the health database search yielded 28,026 results, and the ACM search yielded 541 results. In addition, 2 authors (BP and KB) hand searched the reference lists of related review articles [7,13,18,19,28] and JMIR archives, which returned 17 additional articles. After removing duplicates, 10,017 articles remained.

### Step 3: Study Selection

The study selection was performed by 2 authors (BP and KB) based on inclusion and exclusion criteria to ensure consistency and replicability. As illustrated in Figure 1, we followed the PRISMA (Preferred Reporting Items for Systematic Reviews and Meta-Analyses) procedure [29] to ensure systematic selection. On the basis of the literature review and discussion of the research question, we established several inclusion criteria (Textbox 2). Articles needed to fulfill all the inclusion criteria to be included in the review. After screening the titles of articles, 527 remained for review. The 2 authors independently reviewed the abstracts of each remaining article and, if necessary, downloaded the entire article to check if it fit the criteria. Papers that were potentially eligible were discussed during meetings among the authors. Through these discussions, we also established exclusion criteria (Textbox 2) to disambiguate decisions on potentially relevant articles.

**Inclusion and exclusion criteria for study selection.**

**Inclusion criteria**

1. Articles describe patient-generated photography or videos that reflect personal information and experiences to help address a health and well-being concern
2. Photos or videos are taken by patients, carers, or other participants who are not health care professionals or researchers
3. Findings report on photography or videos as a collection mechanism, intervention, or unit of analysis

**Exclusion criteria**

1. Publications without primary research, such as editorials, opinions, perspectives, reviews, and research protocols
2. Secondary analysis of photos and videos, for example, from social media, that have been shared by individuals without an explicit health or well-being intent
3. Automatic video recordings of consultations or teleconsultations as well as images generated by clinician, surveillance, and patient monitoring systems

On the basis of this process, we selected 110 relevant articles for inclusion in this review. Owing to the large number of papers involved, we only kept track of the number of papers at each stage of the selection process, but we did not record the reason for excluding each paper.

### Step 4: Charting the Data

NVivo (version 12; QSR International), a qualitative data analysis software package, was used to store and manage the charted data. Initially, we charted the data in a predefined form (Multimedia Appendix 3 [15,30-138]), collecting publication data to allow numerical coding and extracting qualitative information relevant to our research questions (eg, author information, year published, aims, target group, research methods, results, number of photos or videos, and values). However, with the large number of articles involved, the diversity of studies, and particularly the breadth of qualitative results presented, the spreadsheet became impractical.

To manage the large volume of data generated through charting, NVivo (version 12) was used to code the content from the PDF version of each article. This process also enabled the next step of collating results. Publication data were extracted verbatim from each article by 2 authors (BP and ARA), whereas coding and critical analysis for the research questions was completed by all authors. Extracted information was discussed at regular meetings of all authors to ensure that the research questions were still relevant, and the articles could answer the research questions and to explore any discrepancies to clarify key concepts and identify major gaps.

### Step 5: Collating, Summarizing, and Reporting Results

Following the recommendation of Arksey and O’Malley [20], we collated and reported the results based on a thematic analysis approach [139] with an analytic framework [140]. Our thematic analysis followed the steps described by Braun and Clarke [139]. We started by reading articles to familiarize ourselves with the data, recorded notes through the *memo* and annotation features of NVivo, and discussed ideas for coding. One author (ARA) manually coded a subset of the 110 articles to generate an initial list of 102 codes relevant to our research questions of health and well-being contexts, value generated, and challenges. These initial codes gave us an overview of the data, but they also highlighted the diversity of study designs and results, which made the aggregation of findings impossible. Instead, we needed a framework to structure and report the results according to our research questions.

To structure the results around health contexts, we initially coded articles according to the International Classification of Diseases, 10th revision [141], a medical classification established by the World Health Organization consisting of 21 chapters. For example, chapter 1 describes infectious and parasitic diseases, which relates to photos and videos used to describe vaccine information and experiences. However, we found that this framework was limited because it presented a medical perspective and did not fit well with articles that reported well-being outcomes or social media contexts. Hence, we revised the structure around the primary contexts presented in the articles: (1) health care services, where patients share images with a health care professional to observe and treat health and well-being concerns; (2) self-management, where patients use images to independently track and manage health concerns; (3) social media, where patients share personal health information and experiences with peers on the web; (4) education, where images are used for health education in schools and waiting rooms; and (5) service improvement, where patients are invited to take images to reflect on their health service experience and express their needs.

To analyze the value of photos and videos reported in our article collection, we used a health consumer engagement framework [140] that highlights six key values of PGHD: functional, emotional, social, transactional, efficiency, and self-determination. For example, the functional value describes how images are used by health care professionals to support health outcomes through diagnosis, explanation, treatment, therapy, and health promotion. The values from this framework were chosen because they originated from a study of patient-generated photos and allowed value to be considered from the perspective of both patients and providers across different health and well-being domains. We chose this framework over benefit-risk models of the health care value, which aim to promote strategic reform [142,143], because photographs and videos are not routinely used in clinical practice, and quantification of value was not demonstrated in the articles retrieved.

To analyze these challenges, we identified several frameworks that describe data challenges [27,144]. Although none of these frameworks captured the range of challenges identified in our initial codes, we selectively applied relevant concepts from these frameworks for our analysis. For example, accessibility is a key challenge for patients [144], which includes lack of access to camera phones, lack of access due to poor app usability, and difficulty in taking photos of feet or the back. From existing frameworks [27,144], we also included the challenges of privacy, interpretability, and relevancy, and we structured the challenges according to different stages of their use: collection, sharing, and examination of photos and videos. In addition, we inductively coded other challenges that emerged from the articles, such as poor photo quality when photos were not in focus or when they did not clearly show the relevant details.

A selection of 15 articles was coded independently by all 3 authors using the chosen frameworks. Regular meetings were held to discuss the suitability of the frameworks for our objectives and to explore any discrepancies in how we applied them in our analysis, especially on how to distinguish between values that appear interrelated (eg, the social and transactional values). Once agreement was reached on how to apply the frameworks and how to structure the challenges, one author (BP) coded the remaining papers. The naming of themes and subthemes was further refined by all the authors while writing the report. The full coding tree is provided in Multimedia Appendix 4. The results present the overall number of articles identified in each theme, as well as the number of articles reporting on photos and videos.

## Results

### Overview

**Figure 2:**
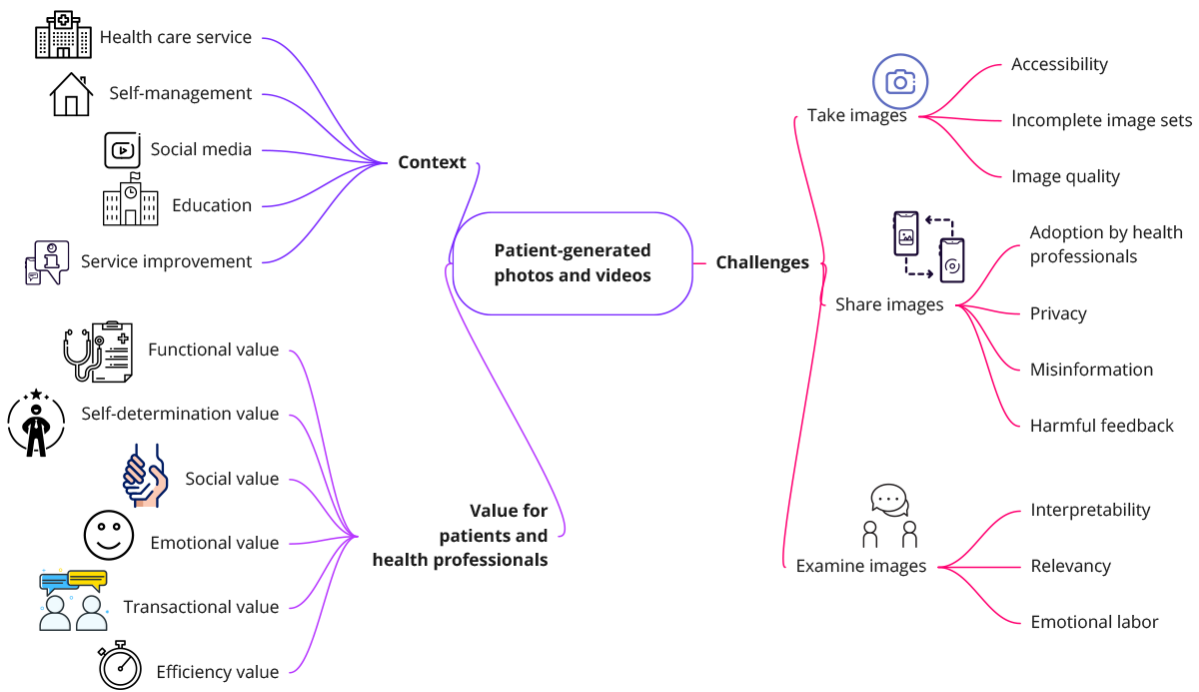
Overview of key themes identified in this review, presenting the contexts in which patients use health photos and videos, the value gained by patients, and challenges experienced.

Of the 110 articles identified in this review, 90 (81.8%) reported on photos, 23 (20.9%) used videos, and 3 (2.7%) used both photos and videos. Figure 2 provides an overview of the key themes revealed in our review, showing the contexts in which photos and videos were used, values gained by patients, and challenges when taking, sharing, and examining photos and videos. The following sections provide further details of each theme.

### Use of Photos and Videos Across Health Contexts

We categorized articles based on the context in which the patient-generated photos and videos were used. As summarized in Table 1, images were largely used in health care services, self-management, and social media contexts. Multimedia Appendix 3 provides a more detailed table that also lists who captured the images (patient or carer), the technologies used to capture and share images, and the audiences receiving them.

**Table 1:** The use of patient-generated photos and videos across health contexts (N=110 articles).^a^

Context	Description	Articles, n (%)	Photos, n (%)	Videos, n (%)	Image information
Health care service	Patients share images with a health care service to observe and treat health and well-being concerns	39 (35.5)	39 (35.5)	2 (1.8)	Skin photos showing potential cancer [30-34], hernia [35], rash [36-38], and wounds [39-50]; foods and beverages consumed [51-62]; experiences related to mental health (eg, death of a parent) [63], emotions such as hope [64], goals for the near future [65] for therapy; and health equipment [66,67] and medication [68]
Self-management	Patients use images to independently track and manage health concerns	33 (30)	33 (30)	1 (0.9)	Foods and beverages consumed [69-92]; nature, people, and events to reflect on emotions [93,94] and lifestyle [95-97]; and smoking and quitting [15,98-100]
Social media	Patients share personal health information and experiences with peers on the web on platforms such as Instagram, Facebook, Flickr, and YouTube	36 (32.7)	19 (17.3)	17 (15.5)	Foods and beverages consumed [88-92]; disease experience (cancer [101-106], cardiovascular [107], diabetes [104,108], kidney stone [109], and multiple sclerosis [110]); mental health (depression [111-113], suicidal thought [114], and other [93,115,116]); proanorexia images [117-120]; medical procedures [121,122]; smoking and quitting [99]; vaccine information [123-127]; and various health concerns [97,128]
Education	Images are used for health education in schools, waiting rooms, and community centers or at home	7 (6.4)	6 (5.5)	1 (0.9)	Digitally altered selfies showing impact of smoking [129,130] and UV exposure [131,132]; healthy eating ideas [133]; toothbrushing behavior videos [134]; and vagina selfies [135]
Service improvement	Patients are invited to take images to reflect on their health service experience and to express their needs	3 (2.7)	1 (0.9)	2 (1.8)	Children’s experiences and challenges in the hospital (eg, needing to process new information) [136,137] and in transitioning to their homes (eg, manage medications) [138]

^a^Several articles reported results on multiple contexts, or they included both photos and videos.

Health care service contexts were described in 35.4% (39/110) of the articles, where patients created photos to document a health concern to share them with a health care professional. The three most common contents in this context were skin photos, food photos, and photos capturing mental health experiences. Patient-generated skin photos were used by dermatologists and general practitioners to review skin lesions and assess potential melanoma [30-34] or rashes [36-38]. Surgeons have also used patient-generated photos to diagnose inguinal hernia [35] and to follow up on surgical wounds or injuries [39-50]. Patient-generated photos showing food and beverages consumed were commonly used by dietitians to support patients with diabetes [54,57,58,60,61], patients with irritable bowel syndrome [53], and pregnant women [51]. Therapists and counselors collaborated with patients to discuss photos capturing events and experiences that affected the patient’s mental health (eg, the death of a parent) [63], emotions such as hope [64], and goals for the near future (eg, to go on a holiday) [65]. In addition, of the 110 studies, 2 (1.8%) used wearable cameras that automatically took photos throughout the day to document dietary intake [55,59], which provided the dietitian with more comprehensive data and alleviated the effort required for patients. Only 1.8% (2/110) of the articles in this context used videos either to document intermittent hand twitching for diagnosis [42] or to record feelings and thoughts about mental health issues between therapy sessions [63].

Self-management contexts accounted for almost a third of the articles (33/110, 30%), where patients used images in their day-to-day lives to track and control a health concern. These images had a clinical or therapeutic context; however, the studies did not report any image sharing with health care services. The most prevalent concern that people self-manage through photos is dietary intake. Food photos provide rich information to recall details of the foods consumed, with whom they were eaten, and the context [74]. People can use food photos to provide accurate energy intake estimates, which do not differ significantly from the gold standard, doubly labeled water, over short periods (6 days) [81]. However, over longer periods (6 months), adherence to photographic food diaries diminishes [73]. Several studies have explored the feasibility of photos with children [69,86] and adolescents [70,71,80,83-85]. In addition to dietary intake, people also use photos of nature, surroundings, people, objects (including foods), and past events to reflect on their emotional state [93,94] or their current lifestyle and well-being [95-97]. Photos are also used by smokers to capture places, events, and routines associated with smoking or quitting cigarettes [15,98-100].

Social media contexts featured in a third of all articles (36/110, 32.7%), where patients share videos and photos with an audience of peers on the web. The summary in Table 1 shows 2 key differences with social media, compared with other contexts. First, almost half of the studies in this context report on videos generated by patients, where they talk about personal health experiences on YouTube. A good example is the study by Liu et al [104], which presents insights from 36 video bloggers who share their experiences with chronic conditions that require self-management, such as diabetes and HIV. The findings show that these videos are often used to teach others about self-management or to keep a personal journal to share their physical and emotional updates in their illness journey. Videos (unlike photos or text alone) allow patients to build rapport with their audiences by filming themselves talking, showing emotions, introducing other people, and showing their health care environments and significant events [104]. A second key difference in the social media context is that images are used to present a broad range of health concerns, including cancer experiences [101-106], mental health [93,111-116], and vaccinations [123-127]. This is partly a result of the focus on experience sharing, where people talk about a disease rather than depict a symptom. It also results from social media, allowing patients to find and join web-based communities dedicated to a shared health concern. A poignant example is proanorexia communities on Flickr, Instagram, and YouTube, which use images and videos to promote eating disorders as a desirable lifestyle rather than as a disease [117-120].

Social media contexts overlapped with self-management contexts (8/110, 7.3% articles), where patients used photos predominantly to self-manage a health concern; however, they also shared these photos with peers on the web. Instagram was used to self-monitor diet [88-92] and emotional well-being [93]. Facebook was used to share photos depicting reasons for quitting cigarettes [99]. A bespoke platform (Staccato) was used to capture and share photos of healthy lifestyle choices such as taking steps instead of an escalator [97].

Educational contexts were described in 6.4% (7/110) of the articles, in which the aim was to educate patients about a health concern. In the school context, a face-aging app was used as an educational intervention to promote smoking cessation [129] and sun protection [131,132]. The app allowed students to take a face selfie and to see the potential impact of smoking cigarettes [129] and UV exposure without sunscreen [131,132] on the way that their face will age. A similar educational intervention has been deployed in the context of a physician’s waiting room to promote smoking cessation [130]. In a home context, *vagina selfies* were used to let women explore and learn about their own intimate anatomy [135], and patient-recorded toothbrushing videos were used to educate dental residents and to refine their toothbrushing behaviors [134].

Finally, health service improvements were described in 2.7% (3/110) of the articles. In this context, health service providers asked patients and their family members to take photos to better understand their patients’ health care experiences with the aim of improving their service delivery. All studies were conducted in pediatric services. Children and parents were invited to take videos or photos to describe their experiences inside the hospital [136,137] and after their transition to their homes [138]. Videos of hospital experiences showed that patients desire better information and ways to share experiences and reflect on feelings [136]. Photos taken at home showed challenges, such as children having to share responsibility for managing medication, and fears and uncertainties, as children adjust to living with a chronic health condition [138].

### The Value of Photos and Videos

Patient-generated photos and videos create significant value when used for health and well-being. On the basis of an engagement framework [140], our analysis identified six key values: functional, self-determination, social, emotional, transactional, and efficiency. Table 2 provides a summary of each value and the number of relevant articles.

**Table 2:** The value of patient-generated photos and videos (N=110 articles).^a^

Value	Description	Articles, n (%)	Photos, n (%)	Videos, n (%)
Functional	Support health outcomes through diagnosis, explanation, treatment, therapy, and health promotion	59 (53.6)	54 (49.1)	7 (6.4)
Self-determination	Empower patient through knowledge, form a personal narrative, and share experiences	39 (35.5)	28 (25.5)	12 (10.9)
Social	Share experience and support with peers, family members, and web-based community members	33 (30)	22 (20)	12 (10.9)
Emotional	Express, understand, and regulate emotions; capture significant moments for therapy	21 (19.1)	18 (16.4)	5 (4.5)
Efficiency	Eliminate unnecessary appointments; replace paper diaries and forms with photographic records	19 (17.3)	19 (17.3)	1 (0.9)
Transactional	Enrich transactions through increased patient engagement and by providing health professionals with a more holistic view of their patients	18 (16.4)	14 (12.7)	6 (5.5)

^a^Several articles reported results on multiple values or on photos and videos.

The most prominent value reported is the functional value (59/110, 53.6%), where photos and videos are used as an aid to support health outcomes through diagnosis, explanation, treatment, therapy, and health promotion. In terms of diagnosis, photos provide important data for health care professionals to diagnose hernias [35], rashes [36], injuries [38], lesions [30,47], and cysts and angioedema [49]. Patients also use photos to self-diagnose skin lesions [32,34] and monitor lesions over time [31]. Photos and videos can provide valuable explanations that lead to new insights for patients about the functioning of their own body [135] and to come to terms with new diagnoses, for example, to cope with cancer [103] and kidney stone disease [109]. For patients with diabetes, photos and videos provide new knowledge about the impact of lifestyle factors such as diet, alcohol consumption, and exercise on their diabetes management [57,58,60,61,104]. Photos can enhance treatment by showing biopsy sites to decrease wrong-site surgery in dermatology [33], medication monitoring [68], and documentation of the healing of postoperative wounds [41,43,45], ulcers [50], and soft-tissue injuries [44]. The therapeutic value of photos and videos was illustrated in reminiscence therapy in patients with Alzheimer disease, where photos were used to support remembering and reminiscing on personal memories [95], as well as in mental health therapy to reflect on past experiences [113]. Finally, photos and videos support health promotion. This is most common with food photos, which help health professionals and patients create an awareness of patterns of eating, food choices, and portion sizes [55,59,62,73,78,82,84-86]; decide on diet changes to promote healthier food choices [37,51,72,76,87]; and aid in weight loss [52,77].

Functional value often went hand in hand with efficiency value (19/110, 17.3%), where the data provided through photos saved time, money, and effort [140]. Commonly reported with photos of skin conditions, time and money are saved when health professionals assess photos instead of assessing patients in person [41,42,44,45] or when patients can self-diagnose skin lesions and rashes [31,32,34,36,40]. Similarly, patients save time and effort when they are allowed to capture their dietary intake through photos, rather than through pen and paper diaries [56,69,71,76,77,79,80,92].

Several values—self-determination, social, and emotional—come from patients using photos and videos to reflect upon, capture, and share personal health experiences, rather than specific data. Self-determination value (39/110, 35.5% articles) arises when patients “confirm and integrate their beliefs (cognitive, spiritual, or other) into health care services, asserting a degree of control over a health care situation congruent with psychological empowerment” [6]. We identified self-determination value from photos and videos through enhanced knowledge, for example, by examining the personal meanings of smoking and related social influences when quitting smoking [15,98,100]. Health professionals sometimes encourage patients to take on more responsibility by monitoring their condition through photos to shift the power in consultations so that patients become more informed and assertive [33,38,39]. Self-determination also arises when patients use videos to form a personal narrative to make sense of a new diagnosis, such as diabetes [58] or cancer [42,101,102], and what is occurring with their bodies, emotions, and social identity before and after medical interventions. Finally, several studies showed self-determination value from sharing personal health experiences, achievements, resources, and advice with other patients through social media [104,133,136]. This was common for mental health conditions, where negative self-perception is a challenge for many patients. In this context, photos can help empower patients through the expression of emotions and negative self-perception as well as through seeing oneself as part of a (web-based) group with same condition [93,112,113,116,117].

Social value (33/110, 30%) comes from sharing health photos and videos with other patients, family members, and friends. Videos are commonly used to share personal experiences and help with others who manage the same illness, for example, diabetes, HIV, cancer, and multiple sclerosis [58,61,101,103,104,106,107,110,121,136]. Patients report that they were motivated to share personal videos because they could not find the web-based information and guidance they wanted [108] and because they gained additional motivation by being able to help other patients [42]. Photo sharing on social media is also common for general healthy living, for example, to share insights about how to eat healthier meals [88-90,133], stay physically active [97], and give up smoking [98,99]. People with mental health conditions also gain value from posting photos on the web to ask questions, call for help, show empathy, and offer support to others [111-114,118,120]. Many patients reported a sense of community with other social media users who are experiencing similar health challenges [67,104,111,112,115,116,119,133].

Emotional value (21/110, 19.1%) can arise from capturing personal experiences with an illness to better understand and regulate emotions [74,93,94,96]. Emotions reported in the studies include a wide range of emotions: sympathy [97], humor [135], hopefulness [64,101,112], fear [101], hopelessness [112], pain [116], suicidal feelings [114], and ambivalent feelings such as simultaneously feeling joyful and worried [98]. Images also allow patients to express emotions and garner support from family members [138], health care providers [136], and web-based audiences [42,102,108,113,114,116]. Patients report that they feel better when they see other social media users who share similar emotions and that photos are more visually stimulating than written text [88]. Therapies involve patient-generated photos to help clients reflect on coping strategies [64,65] and reminisce about past events and emotions [95].

Finally, photos and videos can enrich transactions between patients and health care professionals (18/110, 16.4%). On the one hand, photos can increase patient engagement. Capturing photos together with personal notes helps patients prepare for consultations and take on a more active role in their interactions with health care professionals, for example, by recalling information about their diet [53], skin lesions [46], and experiences with mental illness [65]. People with aphasia can use photos to support expressive communication with health care professionals [66]. On the other hand, patient-generated photos and videos can empower health care professionals. Reviewing photos during consultation can prompt health care professionals to ask questions about health experiences [42,138], triggers for adverse reactions [53], and adherence to treatment plans [42,138]. Photos used in consultations are not always limited to clinical data, as shown in a study with general practitioners who reported that they also see social images (new babies and holidays) that provide them with insights into the broader lives of their patients that impact their health [38]. Photos and videos help health care professionals gain a more holistic view of their patients and empathize better with their patients, for example, in general practice [38], dementia care [95], children’s hospital [136-138], and cancer prevention and treatment [15,102].

### Challenges With Photos and Videos

#### Overview

The final part of our analysis describes the barriers and challenges faced by patients with health-related photographs and videos. Here, our analysis is structured based on the process of working with photos and videos, starting with challenges that patients face when they take photos, when they share them with peers and health professionals, and when they are examined. These challenges are interrelated, meaning that challenges in taking photos and sharing them, in turn, can also affect examination. Table 3 provides a summary of these challenges.

**Table 3:** The challenges faced by patients in taking, sharing, and examining images (N=110 articles).

Challenge			Description		Articles, n (%)		Photos, n (%)		Videos, n (%)
**Image-taking challenges**									
	Accessibility	Lack of access to camera phone; poor app usability; difficulty in taking photos of feet or back		16 (14.5)		16 (14.5)		0 (0)	
	Incomplete image sets	Lapses in food photos over long periods or when people (fail to) reach goal; camera error		23 (20.9)		22 (20)		1 (0.9)	
	Image quality	Image not in focus or not well lit; image not showing relevant details (body part or food)		16 (14.5)		15 (13.6)		1 (0.9)	
**Sharing challenges**									
	Adoption by health professionals	Time and effort required; increased sense of responsibility; limited technical support		4 (3.6)		4 (3.6)		1 (0.9)	
	Privacy	Potential risk to patients and health care professionals captured; lack of safe image transfer; invisible social media audiences		10 (9.1)		8 (7.3)		3 (2.7)	
	Misinformation	Inaccurate or misleading social media images (vaccination); unhealthy behaviors (anorexia)		17 (15.5)		7 (6.4)		10 (9.1)	
	Harmful feedback	Web-based feedback harming people who quit smoking or who share stories of depression		7 (6.4)		4 (3.6)		3 (2.7)	
**Examination challenges**									
	Interpretability	Not enough information in images to assess dietary intake or to diagnose skin lesions		10 (9.1)		10 (9.1)		1 (0.9)	
	Relevancy	Clinicians do not examine images; patients stop when food photos show no new information		6 (5.5)		6 (5.5)		0 (0)	
	Emotional labor	Anxiety about potential infection or cancer diagnosis; stress from revisiting past struggles with surgery or mental illness		7 (6.4)		6 (5.5)		2 (1.8)	

#### Image-Taking Challenges

Image-taking challenges were largely reported with photos. Challenges with capturing videos rarely surfaced in our review, despite the potentially large burden for video (and audio) capture, storage, and editing.

A major challenge in taking images is accessibility (16/110, 14.5%). For example, patients reported difficulty in accessing body parts such as their feet or their backs with a camera phone [30,31] or felt it inappropriate to access their cameras to capture photos of the groin area [31,43,135] and in social situations (to take food photos in public settings such as a restaurant) [54,55,62,74]. Not all patients have access to a camera phone, or they do not know how to use them, particularly children [69] and older patients [33,66]. Usability issues of bespoke apps also limited the accessibility of photos and videos, especially when instructions for taking photos were unclear [33,70,78,135].

In total, 20.9% (23/110) of the articles reported incomplete sets of images as a challenge. This was particularly a concern when patients took photos of their food over long periods [73,74] or when food photos needed to be taken both before and after having a meal to show what has been consumed [55,56,60,69,71,78,79,81,84,85]. Participants reported that the time, effort, and training required to take good images were causes of incomplete sets of data [57,78,82] or simply that they forgot [54,56,57,60,69,71,74,78,82]. People stop taking images when they reach a health goal or when they fail to do so [48,88] or because of life disruptions such as moving to a new home [61].

Patients had difficulties with taking high-quality images (16/110, 14.5%). Photos were not in focus [30,31,33], or photos were too dark to show the relevant body part [31] or food [55,59,82,87]. Often, images did not present all relevant details. For example, photos did not show all ingredients of a meal [60,78,91,133], and videos lacked details on how to complete preparation for a medical procedure [122]. Poor-quality photos of wounds [41,44] and cancer biopsy sites [33] led authors to conclude that patients require further guidance to take high-quality images.

#### Sharing Challenges

Several challenges arise when patients share photos and videos with health professionals and peers. A first barrier is the lack of adoption by health professionals (4/110, 3.6%). Attending to photos and videos takes time and effort [82], with health professionals indicating that they need support from medical assistants to review and identify relevant photos [53]. Adoption is also limited by an increased sense of responsibility for health professionals, for example, when patients assume that health professionals are available all the time and that they take responsibility as soon as photos or videos have been shared [42]. The institutional environment also prohibits adoption, for example, when electronic medical records do not support images taken by patients [45].

Sharing health concerns through photos and videos introduces various privacy risks to patients and their carers (10/110, 9.1%). There is a risk that people may gain access to images on the patient’s phone, for example, patients may accidentally show health photos when showing other images to family and friends [31]. In the context of health services, privacy is at risk when secure and encrypted options for transferring patient photos are not available or when there is a lack of information on who has access to patient photos stored in electronic medical records [45]. Therefore, some clinicians advise their patients to bring photos on their phones instead of sending them, which leaves patients in control and allows them to retain ownership [38]. The privacy of health professionals is also at risk, for example, when patients take images during consultations [38]. Finally, the context of social media introduces privacy risks because the audience is large and unknown, and information can be taken out of context and misinterpreted. For example, videos describing personal experiences with diabetes [108] or memories for people with Alzheimer disease [95] can be seen by not only strangers but also friends and relatives, which can be painful and make them worry.

Misinformation on social media is a common challenge (17/110, 15.5%). This is the only area where videos are more prevalent than photos (10/110, 9.1%, vs 7/110, 6.4% articles). YouTube videos detailing patient experiences can act as a useful source of health information; however, from a medical perspective, these videos can often be inaccurate. For example, patient videos of bowel preparation for colonoscopy often miss important information, such as types of preparation purgatives, disgust, and embarrassment [122]. Videos reporting on breast reconstruction can provide unrealistic expectations [105]. Some videos present unreliable and potentially misleading information about treatments that have no evidence for being effective, such as home remedies for skin cancer [106] and herbal medicines used to treat kidney stone disease [109]. Patient photos and videos posted on social media commonly present vaccinations in a negative light [126], and they receive a higher number of likes than images with positive views toward vaccination [123,124]. Social media are also used to promote harmful behaviors through images of self-injury [116], suicide [114], and eating disorders [117-120].

Patients often share photos and videos on social media to create social value, but such sharing also carries the risk of receiving harmful feedback (7/110, 6.3%). For example, people who quit smoking can gain valuable social support from Facebook groups, but photos posted by current smokers can be counterproductive to quitting attempts [99]. Similarly, people who shared personal experiences with depression [112], rape [115], and thoughts of suicide [114] on social media reported harmful feedback that blamed the victim or even encouraged suicide.

#### Examination Challenges

When patients and health professionals examine photos and videos, a first challenge is interpretability (10/110, 9.1%). The risk of misinterpretation is related to food photos, where photos and accompanying self-reports did not provide sufficient information to accurately assess intake, that is, items of a meal, portion size, and nutritional value, often remained unclear [52,78,80,82,91]. Health professionals expressed concerns about potential misdiagnosis when they rely solely on photos or videos from patients [38,42], and patients also recognize that this is a possibility [31]. Potential misdiagnosis was raised, particularly in the context of skin lesions. Overestimating the significance of a particular lesion may lead to anxiety, but, more importantly, underestimating its significance carries the risk of missed melanoma [30].

A second examination challenge lies in the relevancy of photos and videos (6/110, 5.4%). Consultation times are limited, and health providers do not always see patient photos as relevant enough to examine them [53]. Patients stop taking food photos and sharing food photos on the web when they think they provide no new information and become irrelevant [61,88]. A lack of gender and racial diversity can diminish the relevance of photos and videos on social media for a particular person or target group; for example, they may fail to encourage human papillomavirus vaccination among African American individuals when they do not see themselves represented on the web [127]. Time delays between capturing and examining images can also diminish the relevancy of photos for patients, for example, when reflecting on diet or mental health [87,94].

Finally, the papers also highlighted the challenge of emotional labor, where examining photos triggers emotions that patients and caregivers find difficult to manage (7/110, 6.3%). Photos can add stress to patients, particularly when they already feel stressed from having to manage a chronic illness [137]. Patients also report anxiety about possible health issues raised by photos, such as infection [42] or a cancer diagnosis [34]. Emotional labor can also result from photos that bring back stressful memories from the past, such as an unpleasant surgery or struggles with mental illness [31,65,96]. Revisiting photos from the past was a challenge for people with dementia, as photos used for reminiscing triggered positive emotions of happiness as well as negative emotions of sadness and distress [95].

## Discussion

### Principal Findings

This is the first review to better understand how patient-generated photographs and videos are used across different health and well-being contexts, and what value and challenges they hold for patients and health professionals. In many ways, photos and videos reflect the characteristics of other PGHD; that is, they capture data related to medical conditions or general wellness, are generated by patients or their caregivers, and are often shared with health care professionals, peers, and other stakeholders [1,26]. However, our results highlight several key messages that show that photos and videos are not merely a subset of PGHD but are a powerful medium to engage patients as active partners in their health care, which generates unique value and challenges.

First, photos and videos not only are used in health care services, in education, and for self-management at home but also play an important role in social media contexts. According to the traditional notion of PGHD [1], images offer valuable health data to aid with health decisions in health care services, self-management, and health education. The most common areas in our review were skin photos that assist with the diagnosis of melanoma, food photos to help assess dietary intake, and information related to mental health for discussion with therapists. We also observed images used in unique and unexpected ways. For example, under education, we found that women were invited to take vagina selfies to explore their own intimate anatomy, which can be awkward but helps break associated taboos [135]. Very few studies reported on videos to aid with health decisions, but videos are needed for decisions relating to body movement, such as diagnosing twitching [42] and assessing toothbrushing skills [134]. By contrast, in social media contexts, videos were more common. Instead of presenting data, patient-generated videos (and, to some extent, photos) were used as a medium to communicate personal health knowledge, experiences, and stories to social media audiences. This has also been characterized as health video blogging [104] or visual narrative [116]. Both concepts describe when patients use images simultaneously for personal purposes, particularly to keep a journal and to reminisce, and for communicative purposes, particularly to document their health journey and teach others. Such experience videos are not limited to health concerns that can be easily captured using a camera. Hence, we found a broad range of health and well-being topics discussed on social media, including cancer [104], eating disorders [118,119], and vaccination [123,124].

Second, photos and videos do not only offer functional value to aid with diagnosis and treatment but also provide value to engage and empower patients. On the one hand, the functional value was the most mentioned (59/110, 53.6% articles), where photos (rather than videos) primarily aid with diagnosis, explanation, or treatment. This result aligns with traditional notions of photos as data that offer insights to health providers and patients to address a health concern [1] or even for health providers to monitor patients remotely [144]. On the other hand, our results highlighted several different types of value—self-determination, social, emotional, and transactional —that directly benefit the patient. These types of value arise from active engagement with photos and videos, both through personal reflection (self-determination and emotional values) and when patients interact with health professionals (transactional value) and peers (social value). In particular, self-determination value can lead to patients feeling a higher degree of control in their health care, congruent with psychological empowerment [145]. Overall, our review shows that the different types of value described in the framework of Burns et al [140] are applicable across different health and well-being domains.

Third, although reviews of PGHD emphasize that patients benefit from technologies that reduce the effort required by automatically collecting data such as physical activity, heart rate, and sleep [7,8], our review highlights the opposite: active engagement to record and interpret images is important to generate self-determination value (39/110, 35.5% articles), where patients gain a sense of control over their disease and feel empowered in their health care. Only very few studies explored wearable cameras that automatically take images [55,59,95], but even these studies emphasized the importance of active engagement in reviewing images with caregivers and health professionals. Here, we see a parallel between the papers in our review and visual research methods used in public health, such as photovoice [146,147] and photo-elicitation interviews [148], which show that the effort of representing one’s health through photos pays off because it gives a voice to people that can be empowering [147]. Moreover, visual research methods [146-149] highlight that images can encourage a critical dialogue between different stakeholders to interpret the meaning of an image in a particular social context (eg, a consultation or an online community) and to achieve mutual understanding, which can result in social (33/110, 30%), emotional (21/110, 19.1%), and transactional values (18/110, 16.4%).

Fourth, challenges with photos and videos largely reflect PGHD challenges; however, there are several unique aspects. On the one hand, our results highlight challenges that are reflective of PGHD as discussed in previous work, such as the time and effort required for patients and clinicians [27], incomplete data [27], privacy concerns [7], and limited interpretability and relevancy for clinicians [8,144]. On the other hand, our review highlighted several unique challenges specific to photos and videos. Photos and videos pose unique challenges for data quality, for example, their quality can be diminished by low lighting, lack of focus, and lack of details [30,31,33]. The privacy risks associated with images are potentially higher than those associated with other PGHD because photographic images are more likely to identify the patient than numerical data of physical activity, sleep, and so on. Furthermore, photos and videos are often posted on mainstream social media where privacy is a particular concern, because unlike that in a face-to-face consultation, information on social media is permanent, searchable, copyable, and accessible to invisible audiences [150]. We also found that photos and videos on social media can lead to problematic discourse either through misinformation presented in these images or through harmful feedback from other social media users. These challenges could also affect nonvisual social media data; however, videos allow patients to create a narrative that connects with audiences in ways that are arguably different from numerical or textual health data. For example, video narratives can be persuasive because they personalize information, create dramatic tension, and foster emotional engagement [102], which explains why misinformation was more commonly reported with videos than with photos, despite the smaller number of video articles overall.

Finally, throughout the results, we identified several advantages and disadvantages of photos compared with videos. Photos were more commonly used than videos (90/110, 81.8%, vs 23/110, 20.9% articles) because photos capture the information required to aid decisions in health care service, self-management, and education contexts. Furthermore, photos generally require less effort for capture and examination than videos. However, videos offer a unique advantage through their richness. As explained in media richness theory [151,152], additional details in videos help reduce uncertainty and equivocality for the task at hand. Videos can address uncertainty by providing additional temporal information, which is required to capture and aid with decisions on body movements [42,134] and to provide education on the different steps in a health care procedure [122]. By contrast, equivocality refers to confusion that cannot be clarified by more information but only through a higher quality or richness of information [151,152]. Our review highlighted that such richness in videos was important when patients captured moments of significance, for example, for personal reflection on well-being [96] and for storytelling in therapy sessions [63]. Likewise, such richness was important when patients shared health experiences on social media, which included not only information but also their emotions when dealing with the challenges of cancer [102,104] and mental health disorders [115]. This is not to say that patients cannot use a series of photos and captions to express rich narratives of health experiences on social media, for example, as illustrated by patients using photos to discuss mental health conditions on the web [112]. However, videos provide more opportunities for rich self-expression, for example, through nonverbal cues such as eye contact, facial expressions, and pausing; by involving other actors with their experiences; or by incorporating the physical and temporal contexts of their health experience [104,153].

### Limitations

Our review is subject to several limitations. First, our inclusion criteria limited our review results to only English-language articles and published peer-reviewed literature from 2008 to January 2021.

Second, the articles included in this review comprise diverse study designs, target cohorts, and outcomes. A formal assessment of study quality was not undertaken because this was a scoping review [22], in which most published studies have been pilot or feasibility studies. The review did not find any randomized control trials, which is not surprising because photos and videos are often patient driven.

Third, synthesizing outcomes from a large collection of diverse studies across different contexts was challenging. Only a subset of papers reported health outcomes (reported under the functional value). Many papers presented formative research on the feasibility of introducing photos and videos into a particular context or on the experiences and value gained by patients and health care professionals. Hence, instead of outcomes, we framed the *Results* section more broadly around the various contexts, the value generated for patients and health care professionals, and their challenges. The value for patients and health care professionals was analyzed and collated based on an established framework on the value of PGHD [140]. The analysis of contexts and challenges was largely inductive because existing frameworks for PGHD (eg, the studies by West et al [27] and Abdolkhani et al [144]) did not cover the specifics of photos and videos such as challenges with the photo quality or emotional labor. To ensure consistency, the analysis was conducted independently by 3 members of the research team.

Finally, the broad scope of this review and the large number of articles did not allow for a comparison of effects. On the basis of this scoping review, future work is needed that focuses on specific health domains to critically assess and compare patient outcomes.

### Practical Implications

This review shows that photos and videos provide a powerful way for patients to be actively engaged in their health care. For patients interested in their health, photos taken on smartphones are an accessible means of documenting, sharing, and reflecting on their health, particularly in areas that are easy to photograph, such as diet, skin, and everyday life experiences related to mental health. Patients can also use their phone to film themselves talking and reflecting upon personal experiences relevant to their health and well-being, which is often used to share knowledge on managing chronic conditions [104] or to reflect on experiences affecting their mental health [63,96,115]. Both photos and videos are powerful because they allow patients to share aspects of their health and lived experience, which they cannot easily describe through words alone [15]. Although photos require effort to take and examine, our review shows that such an effort can generate self-determination values where patients feel empowered [140] and that sharing photos can create emotional and social values.

Health care professionals interested in participatory health care [154] can empower patients by encouraging them to take relevant photos and discuss them during consultations. Photos and videos often document important details that a health care professional may not consider asking about [38,42,138]. We have seen that such dialogue about photos and videos can provide transactional value as well as functional value to better diagnose and treat conditions. On the basis of our review, such engagement can be effective when health care professionals are genuinely interested in the data and experiences of their patients to make shared decisions about treatments [155].

Health care professionals and patients must be aware of ethical challenges and professional standards to maintain privacy, confidentiality, and trust [156]. On the one hand, our review shows that health care professionals can build trust by taking the images provided by their patients seriously [38]. On the other hand, image sharing introduces privacy and confidentiality risks through a lack of secure transfer and storage [45], accidental access to other images on a patient’s phone or social media account [31], and potential recordings of the health professional during a consultation [38]. Hence, health care professionals need to be sensitive to and respectful of any patient images to maintain professional relationships and confidentiality [156]. It is recommended that secure platforms be used, for example, by advising patients to bring images on their own phone instead of sharing them via social media [38]. Finally, clear communication is required to inform patients about privacy protection in place and to establish expectations of how images are used [157].

There are two practical implications for health care services. First, health care provider support is crucial for harnessing the power of health data generated through patient photos and videos. Technology infrastructure, training, and policies are needed to safely transfer, store, access, and integrate patient-generated photos and videos with medical records [11]. In addition, health services need to create an environment where their staff has the time and support needed to review and analyze patient data [27,144]. Second, health care services that engage with patients to share photos and videos can gain crucial insights into the patient experience to help them improve their service delivery [136,137].

### Future Research

Scoping reviews are often conducted to determine the value of undertaking a full systematic review [22]. On the basis of the prominent health areas identified in this review, we see value in conducting a narrower review to focus on photos related to skin diseases and to update existing reviews on photos used for dietary assessment [13] and melanoma detection [158]. For dietary assessment, our review identified a large number of recent feasibility studies of food photos with children [69,86] and adolescents [70,71,80,83-85], something that the original systematic review [13] had called for. In addition, our review highlighted that social media play an important role in sharing food photos with peers and gaining social support [88-92]. Similar to an expert review on melanoma detection [158], our results highlighted the importance of patient-generated photos for self-examination and education. In addition, our review also highlighted that patients share melanoma photos and experience videos on social media [101,106]. Finally, our review confirms observations from a professional review on surgical sites [159] that it is feasible for patients to take photos to keep track of wound healing [41,43,45] and that adoption by health professionals remains a challenge due to a lack of time [45].

The breadth of the health areas identified in this review suggests research opportunities to explore patient-generated photos and videos in new health areas. First, in social media contexts, photos and videos are widely used to communicate experiences with diseases that cannot be immediately photographed, such as infectious diseases [123-125,127], Alzheimer disease [95], and myocardial infarction [107]. This breadth suggests that photos and videos can also offer value with other health and well-being contexts that may be invisible to the camera but can be discussed, such as back pain, arthritis, and other musculoskeletal conditions. Second, in the context of education and health promotion, the study of vagina selfies, which allows women to explore their own intimate anatomy [135], as well as the use of videos to share experiences with mammography [121], suggests a broader potential of photos and videos to reflect on women’s health, for example, with pregnancy and childbirth, osteoporosis, or breast cancer. Third, in the contexts of health care services, we see potential for using videos more widely to capture body movements for clinical purposes, similar to the presented studies on capturing toothbrushing skills [134] and an intermittently twitching hand [42]. For example, consultations with physiotherapists could benefit from patient-generated videos that capture rehabilitation exercises and activities of daily living at the patient’s home.

The challenges faced by patients identified in this review highlight the need for further research on technological design. More work is needed to better understand accessibility needs, particularly when capturing videos in a health context. Collaboration between patients and caregivers is needed to ensure that technologies are usable and accessible. To encourage patients to take images, research into protocols and technology designs that train patients to take high-quality photos as well as provide relevant medical knowledge is needed [57,78,160]. To ensure high-quality images, newer smartphone cameras that offer higher sensitivity in low-light settings need to be harnessed together with research into designing visual aids and voice feedback to guide users in taking photos that capture the required content [28,161]. Finally, to retain engagement, patients benefit from technology designs that assist them in examining their images more effectively. This involves highlighting relevant information in photos, as well as integrating photos with other data that might be scattered across other devices, such as vital signs and lifestyle data from mobile and wearable devices, to explore connections and trends across different data sources [8].

The identified interpretability challenges highlight the need for further research to enhance the relevancy of photos and videos for clinicians. On the one hand, empirical research is needed to better understand the goals and priorities of clinicians [8]. On the other hand, sociotechnical studies are needed to explore how emerging technologies such as machine learning techniques can be harnessed to better manage the large number of photographic images. Our review included only 1 study that examined machine learning techniques to aid in melanoma detection in patient-generated photos [34], whereas in medical imaging, machine learning techniques are already used in clinical practice to aid in the diagnosis and prognosis of various health concerns [162,163]. However, even with sophisticated machine learning algorithms, effective integration into clinical practice remains an open question [164,165].

Finally, more research is needed to investigate privacy and misinformation on social media [166]. The privacy of PGHD is a complex issue across many forms of PGHD [1,7,11], which cannot be addressed simply through a more secure technology infrastructure or privacy policies. Inspired by Palen and Dourish [167], we see privacy as a dynamic practice in which patients negotiate access to personal information according to circumstances. More research is needed to investigate how patients manage their privacy under different circumstances: when they capture photos, manage them on their phones, share them in consultations, or post them on social media. Furthermore, our review identified that patient photos and videos shared on social media provide inaccurate and sometimes misleading information on vaccinations [123,124,126]. In light of current efforts to provide COVID-19 vaccines throughout the world, further research is needed to understand the dangers of misinformation on social media and their impact on public health advice on the COVID-19 pandemic and vaccinations, as well as research to harness social media to improve the health literacy of patients [168].

### Conclusions

This review showed that patient-generated photos and videos are used across a wide range of health care activities. Similar to other forms of PGHD, photos and videos provide critical information to aid in the diagnosis and treatment of various health conditions. However, going beyond textual and numerical PGHD, photos and videos are powerful media that facilitate rich and meaningful interactions, both in person and on social media. They connect fellow patients and facilitate the exchange of social and emotional support. Photos and videos are also powerful media for enriching transactions with health care professionals. Ultimately, they engage patients with their own health and well-being and empower them in their own care.

On the basis of this review, we present agenda for future research. On the one hand, this review highlighted opportunities to expand the use of photos and videos to other health and well-being areas and to better implement them in clinical practice. On the other hand, this review raised the need for more research to address key challenges such as accessibility for patients, relevancy and interpretability for clinicians, and privacy and misinformation on social media, to fully realize the potential of patient-generated photos and videos for health and well-being.

## Appendix

Multimedia Appendix 1 PRISMA-ScR (Preferred Reporting Items for Systematic Reviews and Meta-Analyses extension for Scoping Reviews) checklist.

Multimedia Appendix 2 Search strategy and keywords for all databases.

Multimedia Appendix 3 Data extraction spreadsheet.

Multimedia Appendix 4 Coding tree, exported from NVivo (version 12; QSR International).

## Abbreviations

PGHD: patient-generated health data
PRISMA: Preferred Reporting Items for Systematic Reviews and Meta-Analyses
PROM: patient-reported outcome measure

## References

[1] Shapiro M, Johnston D, Wald J, Mon D Patient-generated health data. Health IT.

[2] Klasnja P, Pratt W (2012). Healthcare in the pocket: mapping the space of mobile-phone health interventions. J Biomed Inform.

[3] Dennison L, Morrison L, Conway G, Yardley L (2013). Opportunities and challenges for smartphone applications in supporting health behavior change: qualitative study. J Med Internet Res.

[4] Loncar-Turukalo T, Zdravevski E, Machado da Silva J, Chouvarda I, Trajkovik V (2019). Literature on wearable technology for connected health: scoping review of research trends, advances, and barriers. J Med Internet Res.

[5] Bragazzi NL (2013). From P0 to P6 medicine, a model of highly participatory, narrative, interactive, and "augmented" medicine: some considerations on Salvatore Iaconesi's clinical story. Patient Prefer Adherence.

[6] Burns K Engagement, empowerment and patient generated health data. Queensland University of Technology (QUT).

[7] Nittas V, Lun P, Ehrler F, Puhan MA, Mütsch M (2019). Electronic patient-generated health data to facilitate disease prevention and health promotion: scoping review. J Med Internet Res.

[8] Choe EK, Lee B, Andersen TO, Wilcox L, Fitzpatrick G (2018). Harnessing the power of patient-generated data. IEEE Pervasive Comput.

[9] Lavallee DC, Lee JR, Austin E, Bloch R, Lawrence SO, McCall D, Munson SA, Nery-Hurwit MB, Amtmann D (2020). mHealth and patient generated health data: stakeholder perspectives on opportunities and barriers for transforming healthcare. Mhealth.

[10] Black N (2013). Patient reported outcome measures could help transform healthcare. BMJ.

[11] Demiris G, Iribarren SJ, Sward K, Lee S, Yang R (2019). Patient generated health data use in clinical practice: a systematic review. Nurs Outlook.

[12] Burns K (2015). Digital photography and the medical selfie. J Particip Med.

[13] Gemming L, Utter J, Ni Mhurchu C (2015). Image-assisted dietary assessment: a systematic review of the evidence. J Acad Nutr Diet.

[14] Berger J (1972). Ways of Seeing.

[15] Haines RJ, Oliffe JL, Bottorff JL, Poland BD (2010). 'The missing picture': tobacco use through the eyes of smokers. Tob Control.

[16] Wicks P, Massagli M, Frost J, Brownstein C, Okun S, Vaughan T, Bradley R, Heywood J (2010). Sharing health data for better outcomes on PatientsLikeMe. J Med Internet Res.

[17] Liu L, Gu J, Shao F, Liang X, Yue L, Cheng Q, Zhang L (2020). Application and preliminary outcomes of remote diagnosis and treatment during the COVID-19 outbreak: retrospective cohort study. JMIR Mhealth Uhealth.

[18] Boulton NE, Williams J, Jones RS (2018). Could participant-produced photography augment therapeutic interventions for people with intellectual disabilities? A systematic review of the available evidence. J Intellect Disabil.

[19] Mason RA, Davis HS, Boles MB, Goodwyn F (2013). Efficacy of point-of-view video modeling. Remedial Special Educ.

[20] Arksey H, O'Malley L (2005). Scoping studies: towards a methodological framework. Int J Soc Res Methodol.

[21] Grant M, Booth A (2009). A typology of reviews: an analysis of 14 review types and associated methodologies. Health Info Libr J.

[22] Levac D, Colquhoun H, O'Brien KK (2010). Scoping studies: advancing the methodology. Implement Sci.

[23] Davis K, Drey N, Gould D (2009). What are scoping studies? A review of the nursing literature. Int J Nurs Stud.

[24] Tricco AC, Lillie E, Zarin W, O'Brien KK, Colquhoun H, Levac D, Moher D, Peters MD, Horsley T, Weeks L, Hempel S, Akl EA, Chang C, McGowan J, Stewart L, Hartling L, Aldcroft A, Wilson MG, Garritty C, Lewin S, Godfrey CM, Macdonald MT, Langlois EV, Soares-Weiser K, Moriarty J, Clifford T, Tunçalp Ö, Straus SE (2018). PRISMA extension for scoping reviews (PRISMA-ScR): checklist and explanation. Ann Intern Med.

[25] Ray A, Scott A, Nikkhah D, Dheansa B (2015). The medical selfie. BMJ.

[26] Figueiredo MC, Chen Y (2020). Patient-generated health data: dimensions, challenges, and open questions. FNT Human Comput Interact.

[27] West P, Kleek MV, Giordano R, Weal MJ, Shadbolt N (2018). Common barriers to the use of patient-generated data across clinical settings. Proceedings of the 2018 CHI Conference on Human Factors in Computing Systems.

[28] Brown R, Ploderer B, Seng L, Lazzarini P, Van Netten J (2017). MyFootCare: a mobile self-tracking tool to promote self-care amongst people with diabetic foot ulcers. Proceedings of the 29th Australian Conference on Computer-Human Interaction.

[29] Moher D, Liberati A, Tetzlaff J, Altman DG, PRISMA Group (2009). Preferred reporting items for systematic reviews and meta-analyses: the PRISMA statement. PLoS Med.

[30] Boyce Z, Gilmore S, Xu C, Soyer H (2011). The remote assessment of melanocytic skin lesions: a viable alternative to face-to-face consultation. Dermatology.

[31] Diethei D, Colley A, Kalving M, Salmela T, Häkkilä J, Schöning J (2020). Medical selfies: emotional impacts and practical challenges. Proceedings of the 22nd International Conference on Human-Computer Interaction with Mobile Devices and Services.

[32] Kantor J (2015). Skin self-photography for dysplastic nevus monitoring is associated with a decrease in the number of biopsies at follow-up: a retrospective analytical study. J Am Acad Dermatol.

[33] Nijhawan R, Lee E, Nehal K (2015). Biopsy site selfies--a quality improvement pilot study to assist with correct surgical site identification. Dermatol Surg.

[34] Zhao J, Cheung N, Sosa R, Koh D (2015). Design self-diagnosis applications for non-patients. Proceedings of the 33rd Annual ACM Conference Extended Abstracts on Human Factors in Computing Systems.

[35] Saleem M (2008). Digital imaging by parents: an aid to the diagnosis of inguinal hernia in infants and children. Singapore Med J.

[36] Hartgers M, Jatoi A (2010). E-mail and photographs: a case report of a patient-initiated diagnostic tool in the era of electronic communication. J Palliat Med.

[37] Ginting K, Stolfi A, Wright J, Omoloja A (2020). Patient portal, patient-generated images, and medical decision-making in a pediatric ambulatory setting. Appl Clin Inform.

[38] Tan L, Hu W, Brooker R (2014). Patient-initiated camera phone images in general practice: a qualitative study of illustrated narratives. Br J Gen Pract.

[39] Frühauf J, Schwantzer G, Ambros-Rudolph CM, Weger W, Ahlgrimm-Siess V, Salmhofer W, Hofmann-Wellenhof R (2010). Pilot study using teledermatology to manage high-need patients with psoriasis. Arch Dermatol.

[40] Frühauf J, Schwantzer G, Ambros-Rudolph CM, Weger W, Ahlgrimm-Siess V, Salmhofer W, Hofmann-Wellenhof R (2012). Pilot study on the acceptance of mobile teledermatology for the home monitoring of high-need patients with psoriasis. Australas J Dermatol.

[41] Kummerow Broman K, Oyefule OO, Phillips SE, Baucom RB, Holzman MD, Sharp KW, Pierce RA, Nealon WH, Poulose BK (2015). Postoperative care using a secure online patient portal: changing the (inter)face of general surgery. J Am Coll Surg.

[42] Burns K, McBride CA, Patel B, FitzGerald G, Mathews S, Drennan J (2019). Creating consumer-generated health data: interviews and a pilot trial exploring how and why patients engage. J Med Internet Res.

[43] Gunter R, Fernandes-Taylor S, Mahnke A, Awoyinka L, Schroeder C, Wiseman J, Sullivan S, Bennett K, Greenberg C, Kent KC (2016). Evaluating patient usability of an image-based mobile health platform for postoperative wound monitoring. JMIR Mhealth Uhealth.

[44] Michiels E, Ouellette L, Bush C, VanDePol E, Fleeger T, Jones JS (2017). Camera phones for the follow-up of soft-tissue injuries in adult and pediatric ED patients: a feasibility study. Am J Emerg Med.

[45] Miller M, Ross R, Voight C, Brouwer H, Karavite D, Gerber J, Grundmeier R, Coffin S (2017). Patient-generated digital images after pediatric ambulatory surgery. Appl Clin Inform.

[46] Mishra S, Miller A, Haldar S, Khelifi M, Eschler J, Elera R, Pollack A, Pratt W (2018). Supporting collaborative health tracking in the hospital: patients' perspectives. Proceedings of the 2018 CHI Conference on Human Factors in Computing Systems.

[47] Pourdanesh F, Sayyedi A, Jamilian A, Yaghmaei M (2012). Application of self-recorded photos using mobile phones in maxillofacial surgery. J MTM.

[48] Walker TW, O'Connor N, Byrne S, McCann PJ, Kerin MJ (2011). Electronic follow-up of facial lacerations in the emergency department. J Telemed Telecare.

[49] Hanu-Cernat LM, Hall E, Barnard NA (2009). The diagnostic value of clinical photographs taken by patients. Br J Oral Maxillofac Surg.

[50] Dinsdale G, Moore T, Manning J, Murray A, Atkinson R, Ousey K, Dickinson M, Taylor C, Herrick AL (2018). Tracking digital ulcers in systemic sclerosis: a feasibility study assessing lesion area in patient-recorded smartphone photographs. Ann Rheum Dis.

[51] Ashman AM, Collins CE, Brown LJ, Rae KM, Rollo ME (2016). A brief tool to assess image-based dietary records and guide nutrition counselling among pregnant women: an evaluation. JMIR Mhealth Uhealth.

[52] Carter MC, Burley VJ, Nykjaer C, Cade JE (2013). 'My Meal Mate' (MMM): validation of the diet measures captured on a smartphone application to facilitate weight loss. Br J Nutr.

[53] Chung C, Wang Q, Schroeder J, Cole A, Zia J, Fogarty J, Munson SA (2019). Identifying and planning for individualized change: patient-provider collaboration using lightweight food diaries in healthy eating and irritable bowel syndrome. Proc ACM Interact Mob Wearable Ubiquitous Technol.

[54] Ehrmann BJ, Anderson RM, Piatt GA, Funnell MM, Rashid H, Shedden K, Douyon L (2014). Digital photography as an educational food logging tool in obese patients with type 2 diabetes: lessons learned from a randomized, crossover pilot trial. Diabetes Educ.

[55] Gemming L, Doherty A, Kelly P, Utter J, Ni Mhurchu C (2013). Feasibility of a SenseCam-assisted 24-h recall to reduce under-reporting of energy intake. Eur J Clin Nutr.

[56] Higgins JA, LaSalle AL, Zhaoxing P, Kasten MY, Bing KN, Ridzon SE, Witten TL (2009). Validation of photographic food records in children: are pictures really worth a thousand words?. Eur J Clin Nutr.

[57] Karway G, Grando MA, Grimm K, Groat D, Cook C, Thompson B (2020). Self-management behaviors of patients with type 1 diabetes: comparing two sources of patient-generated data. Appl Clin Inform.

[58] Mamykina L, Miller A, Mynatt E, Greenblatt D (2010). Constructing identities through storytelling in diabetes management. Proceedings of the SIGCHI Conference on Human Factors in Computing Systems.

[59] O'Loughlin G, Cullen SJ, McGoldrick A, O'Connor S, Blain R, O'Malley S, Warrington GD (2013). Using a wearable camera to increase the accuracy of dietary analysis. Am J Prev Med.

[60] Rollo ME, Ash S, Lyons-Wall P, Russell A (2011). Trial of a mobile phone method for recording dietary intake in adults with type 2 diabetes: evaluation and implications for future applications. J Telemed Telecare.

[61] Sun S, Belkin NJ (2016). Managing personal information over the long-term, or not? Experiences by type 1 diabetes patients. Proc Assoc Info Sci Tech.

[62] Ptomey LT, Willis EA, Goetz JR, Lee J, Sullivan DK, Donnelly JE (2015). Digital photography improves estimates of dietary intake in adolescents with intellectual and developmental disabilities. Disabil Health J.

[63] Matthews M, Doherty G (2011). My mobile story: therapeutic storytelling for children. Proceedings of the CHI '11 Extended Abstracts on Human Factors in Computing Systems.

[64] Quaglietti S (2020). Creating a hope narrative for veterans in recovery using photography and written expression. J Creat Ment Health.

[65] Sitvast JE, Abma TA, Widdershoven GA (2010). Facades of suffering: clients' photo stories about mental illness. Arch Psychiatr Nurs.

[66] Ulmer E, Hux K, Brown J, Nelms T, Reeder C (2016). Using self-captured photographs to support the expressive communication of people with aphasia. Aphasiology.

[67] Waycott J, Vetere F, Pedell S, Kulik L, Ozanne E, Gruner A, Downs J (2013). Older adults as digital content producers. Proceedings of the SIGCHI Conference on Human Factors in Computing Systems.

[68] Galloway G, Coyle J, Guillén JE, Flower K, Mendelson J (2011). A simple, novel method for assessing medication adherence: capsule photographs taken with cellular telephones. J Addict Med.

[69] Aflague TF, Boushey CJ, Guerrero RT, Ahmad Z, Kerr DA, Delp EJ (2015). Feasibility and use of the mobile food record for capturing eating occasions among children ages 3-10 years in Guam. Nutrients.

[70] Boushey CJ, Harray AJ, Kerr DA, Schap TE, Paterson S, Aflague T, Bosch Ruiz M, Ahmad Z, Delp EJ (2015). How willing are adolescents to record their dietary intake? The mobile food record. JMIR Mhealth Uhealth.

[71] Casperson SL, Sieling J, Moon J, Johnson L, Roemmich JN, Whigham L (2015). A mobile phone food record app to digitally capture dietary intake for adolescents in a free-living environment: usability study. JMIR Mhealth Uhealth.

[72] Coary S, Poor M (2016). How consumer-generated images shape important consumption outcomes in the food domain. J Consum Market.

[73] Comulada WS, Swendeman D, Koussa MK, Mindry D, Medich M, Estrin D, Mercer N, Ramanathan N (2018). Adherence to self-monitoring healthy lifestyle behaviours through mobile phone-based ecological momentary assessments and photographic food records over 6 months in mostly ethnic minority mothers. Public Health Nutr.

[74] Cordeiro F, Bales E, Cherry E, Fogarty J (2015). Rethinking the mobile food journal: exploring opportunities for lightweight photo-based capture. Proceedings of the 33rd Annual ACM Conference on Human Factors in Computing Systems.

[75] Daugherty BL, Schap TE, Ettienne-Gittens R, Zhu FM, Bosch M, Delp EJ, Ebert DS, Kerr DA, Boushey CJ (2012). Novel technologies for assessing dietary intake: evaluating the usability of a mobile telephone food record among adults and adolescents. J Med Internet Res.

[76] Doumit R, Long J, Kazandjian C, Gharibeh N, Karam L, Song H, Boswell C, Zeeni N (2016). Effects of recording food intake using cell phone camera pictures on energy intake and food choice. Worldviews Evid Based Nurs.

[77] Dunn CG, Turner-McGrievy GM, Wilcox S, Hutto B (2019). Dietary self-monitoring through calorie tracking but not through a digital photography app is associated with significant weight loss: the 2smart pilot study-a 6-month randomized trial. J Acad Nutr Diet.

[78] Fowler LA, Yingling LR, Brooks AT, Wallen GR, Peters-Lawrence M, McClurkin M, Wiley KL, Mitchell VM, Johnson TD, Curry KE, Johnson AA, Graham AP, Graham LA, Powell-Wiley TM (2018). Digital food records in community-based interventions: mixed-methods pilot study. JMIR Mhealth Uhealth.

[79] Hongu N, Pope BT, Bilgiç P, Orr BJ, Suzuki A, Kim AS, Merchant NC, Roe DJ (2015). Usability of a smartphone food picture app for assisting 24-hour dietary recall: a pilot study. Nutr Res Pract.

[80] Lee CD, Chae J, Schap TE, Kerr DA, Delp EJ, Ebert DS, Boushey CJ (2012). Comparison of known food weights with image-based portion-size automated estimation and adolescents' self-reported portion size. J Diabetes Sci Technol.

[81] Martin CK, Correa JB, Han H, Allen HR, Rood JC, Champagne CM, Gunturk BK, Bray GA (2012). Validity of the Remote Food Photography Method (RFPM) for estimating energy and nutrient intake in near real-time. Obesity (Silver Spring).

[82] Martin CK, Han H, Coulon SM, Allen HR, Champagne CM, Anton SD (2009). A novel method to remotely measure food intake of free-living individuals in real time: the remote food photography method. Br J Nutr.

[83] Ptomey LT, Willis EA, Honas JJ, Mayo MS, Washburn RA, Herrmann SD, Sullivan DK, Donnelly JE (2015). Validity of energy intake estimated by digital photography plus recall in overweight and obese young adults. J Acad Nutr Diet.

[84] Segovia-Siapco G, Sabaté J (2016). Using personal mobile phones to assess dietary intake in free-living adolescents: comparison of face-to-face versus telephone training. JMIR Mhealth Uhealth.

[85] Six BL, Schap TE, Zhu FM, Mariappan A, Bosch M, Delp EJ, Ebert DS, Kerr DA, Boushey CJ (2010). Evidence-based development of a mobile telephone food record. J Am Diet Assoc.

[86] Small L, Sidora-Arcoleo K, Vaughan L, Creed-Capsel J, Chung K, Stevens C (2009). Validity and reliability of photographic diet diaries for assessing dietary intake among young children. ICAN Infant Child Adolescent Nutrition.

[87] Zepeda L, Deal D (2008). Think before you eat: photographic food diaries as intervention tools to change dietary decision making and attitudes. Int J Consum Stud.

[88] Chung C, Agapie E, Schroeder J, Mishra S, Fogarty J, Munson S (2017). When personal tracking becomes social: examining the use of Instagram for healthy eating. Proceedings of the 2017 CHI Conference on Human Factors in Computing Systems.

[89] Epstein D, Cordeiro F, Fogarty J, Hsieh G, Munson S (2016). Crumbs: lightweight daily food challenges to promote engagement and mindfulness. Proceedings of the 2016 CHI Conference on Human Factors in Computing Systems.

[90] Helander E, Kaipainen K, Korhonen I, Wansink B (2014). Factors related to sustained use of a free mobile app for dietary self-monitoring with photography and peer feedback: retrospective cohort study. J Med Internet Res.

[91] Linehan C, Doughty M, Lawson S, Kirman B, Olivier P, Moynihan P (2010). Tagliatelle: social tagging to encourage healthier eating. Proceedings of the CHI '10 Extended Abstracts on Human Factors in Computing Systems.

[92] Turner-McGrievy GM, Helander EE, Kaipainen K, Perez-Macias JM, Korhonen I (2015). The use of crowdsourcing for dietary self-monitoring: crowdsourced ratings of food pictures are comparable to ratings by trained observers. J Am Med Inform Assoc.

[93] Nave C, Romão T, Correia N (2018). Self-tracking emotional states through social media mobile photography. Proceedings of the 32nd International BCS Human Computer Interaction Conference (HCI).

[94] Tsujita H, Rekimoto J (2011). HappinessCounter: smile-encouraging appliance to increase positive mood. Proceedings of the CHI '11 Extended Abstracts on Human Factors in Computing Systems.

[95] Crete-Nishihata M, Baecker R, Massimi M, Ptak D, Campigotto R, Kaufman L, Brickman A, Turner G, Steinerman J, Black S (2012). Reconstructing the past: personal memory technologies are not just personal and not just for memory. Human Comput Interact.

[96] Isaacs E, Konrad A, Walendowski A, Lennig T, Hollis V, Whittaker S (2013). Echoes from the past: how technology mediated reflection improves well-being. Proceedings of the SIGCHI Conference on Human Factors in Computing Systems.

[97] Adams P, Baumer E, Gay G (2014). Staccato social support in mobile health applications. Proceedings of the SIGCHI Conference on Human Factors in Computing Systems.

[98] Haines-Saah RJ, Oliffe JL, White CF, Bottorff JL (2013). "It is just not part of the culture here": young adults' photo-narratives about smoking, quitting, and healthy lifestyles in Vancouver, Canada. Health Place.

[99] Haines-Saah RJ, Kelly MT, Oliffe JL, Bottorff JL (2015). Picture Me Smokefree: a qualitative study using social media and digital photography to engage young adults in tobacco reduction and cessation. J Med Internet Res.

[100] Oliffe J, Bottorff J, Kelly M, Halpin M (2008). Analyzing participant produced photographs from an ethnographic study of fatherhood and smoking. Res Nurs Health.

[101] Cho H, Silver N, Na K, Adams D, Luong KT, Song C (2018). Visual cancer communication on social media: an examination of content and effects of #Melanomasucks. J Med Internet Res.

[102] Chou WS, Hunt Y, Folkers A, Augustson E (2011). Cancer survivorship in the age of YouTube and social media: a narrative analysis. J Med Internet Res.

[103] Clerici C, Veneroni L, Bisogno G, Trapuzzano A, Ferrari A (2012). Videos on rhabdomyosarcoma on YouTube: an example of the availability of information on pediatric tumors on the web. J Pediatr Hematol Oncol.

[104] Liu L, Huh J, Neogi T, Inkpen K, Pratt W (2013). Health vlogger-viewer interaction in chronic illness management. Proceedings of the SIGCHI Conference on Human Factors in Computing Systems.

[105] Tan ML, Kok K, Ganesh V, Thomas SS (2014). Patient information on breast reconstruction in the era of the world wide web. A snapshot analysis of information available on youtube.com. Breast.

[106] Basch CH, Basch CE, Hillyer GC, Reeves R (2015). YouTube videos related to skin cancer: a missed opportunity for cancer prevention and control. JMIR Cancer.

[107] Pant S, Deshmukh A, Murugiah K, Kumar G, Sachdeva R, Mehta JL (2012). Assessing the credibility of the "YouTube approach" to health information on acute myocardial infarction. Clin Cardiol.

[108] Gómez-Zúñiga B, Fernandez-Luque L, Pousada M, Hernández-Encuentra E, Armayones M (2012). ePatients on YouTube: analysis of four experiences from the patients' perspective. Med 2 0.

[109] Sood A, Sarangi S, Pandey A, Murugiah K (2011). YouTube as a source of information on kidney stone disease. Urology.

[110] Fernandez-Luque L, Elahi N, Grajales F (2009). An analysis of personal medical information disclosed in YouTube videos created by patients with multiple sclerosis. Studies in Health Technology and Informatics.

[111] Andalibi N, Ozturk P, Forte A (2015). Depression-related imagery on instagram. Proceedings of the 18th ACM Conference Companion on Computer Supported Cooperative Work & Social Computing.

[112] Andalibi N, Ozturk P, Forte A (2017). Sensitive self-disclosures, responses, and social support on Instagram: the case of #Depression. Proceedings of the 2017 ACM Conference on Computer Supported Cooperative Work and Social Computing.

[113] Manikonda L, De Choudhury M (2017). Modeling and understanding visual attributes of mental health disclosures in social media. Proceedings of the 2017 CHI Conference on Human Factors in Computing Systems.

[114] Brown RC, Bendig E, Fischer T, Goldwich AD, Baumeister H, Plener PL (2019). Can acute suicidality be predicted by Instagram data? Results from qualitative and quantitative language analyses. PLoS One.

[115] Harrington C (2018). Neo-liberal subjectivity, self-branding and ‘my rape story’ YouTube videos. Crit Sociol.

[116] Seko Y (2013). Picturesque wounds: a multimodal analysis of self-injury photographs on flickr. Forum Qual Soc Res.

[117] Chancellor S, Lin Z, Goodman E, Zerwas S, De Choudhury M (2016). Quantifying and predicting mental illness severity in online pro-eating disorder communities. Proceedings of the 19th ACM Conference on Computer-Supported Cooperative Work & Social Computing.

[118] Oksanen A, Garcia D, Sirola A, Näsi M, Kaakinen M, Keipi T, Räsänen P (2015). Pro-anorexia and anti-pro-anorexia videos on YouTube: sentiment analysis of user responses. J Med Internet Res.

[119] Syed-Abdul S, Fernandez-Luque L, Jian W, Li Y, Crain S, Hsu M, Wang Y, Khandregzen D, Chuluunbaatar E, Nguyen PA, Liou D (2013). Misleading health-related information promoted through video-based social media: anorexia on YouTube. J Med Internet Res.

[120] Yom-Tov E, Fernandez-Luque L, Weber I, Crain SP (2012). Pro-anorexia and pro-recovery photo sharing: a tale of two warring tribes. J Med Internet Res.

[121] Basch CH, Hillyer GC, MacDonald ZL, Reeves R, Basch CE (2015). Characteristics of YouTube videos related to mammography. J Cancer Educ.

[122] Basch CH, Hillyer GC, Reeves R, Basch CE (2014). Analysis of YouTube videos related to bowel preparation for colonoscopy. World J Gastrointest Endosc.

[123] Ache KA, Wallace LS (2008). Human papillomavirus vaccination coverage on YouTube. Am J Prev Med.

[124] Briones R, Nan X, Madden K, Waks L (2012). When vaccines go viral: an analysis of HPV vaccine coverage on YouTube. Health Commun.

[125] Chen T, Dredze M (2018). Vaccine images on Twitter: analysis of what images are shared. J Med Internet Res.

[126] Guidry JP, Carlyle K, Messner M, Jin Y (2015). On pins and needles: how vaccines are portrayed on Pinterest. Vaccine.

[127] Lama Y, Chen T, Dredze M, Jamison A, Quinn SC, Broniatowski DA (2018). Discordance between human papillomavirus Twitter images and disparities in human papillomavirus risk and disease in the United States: mixed-methods analysis. J Med Internet Res.

[128] Choe E, Lee N, Lee B, Pratt W, Kientz J (2014). Understanding quantified-selfers' practices in collecting and exploring personal data. Proceedings of the SIGCHI Conference on Human Factors in Computing Systems.

[129] Brinker T, Alfitian J, Seeger W, Groneberg D, von Kalle C, Enk A, Herth F, Kreuter M, Bauer C, Gatzka M, Suhre J (2018). A face-aging smoking prevention/cessation intervention for nursery school students in Germany: an appearance-focused interventional study. Int J Environ Res Public Health.

[130] Brinker TJ, Brieske CM, Esser S, Klode J, Mons U, Batra A, Rüther T, Seeger W, Enk AH, von Kalle C, Berking C, Heppt MV, Gatzka MV, Bernardes-Souza B, Schlenk RF, Schadendorf D (2018). A face-aging app for smoking cessation in a waiting room setting: pilot study in an HIV outpatient clinic. J Med Internet Res.

[131] Brinker TJ, Brieske CM, Schaefer CM, Buslaff F, Gatzka M, Petri MP, Sondermann W, Schadendorf D, Stoffels I, Klode J (2017). Photoaging mobile apps in school-based melanoma prevention: pilot study. J Med Internet Res.

[132] Brinker TJ, Heckl M, Gatzka M, Heppt MV, Resende Rodrigues H, Schneider S, Sondermann W, de Almeida E Silva C, Kirchberger MC, Klode J, Enk AH, Knispel S, von Kalle C, Stoffels I, Schadendorf D, Nakamura Y, Esser S, Assis A, Bernardes-Souza B (2018). A skin cancer prevention facial-aging mobile app for secondary schools in Brazil: appearance-focused interventional study. JMIR Mhealth Uhealth.

[133] Parker A, Kantroo V, Lee H, Osornio M, Sharma M, Grinter R (2012). Health promotion as activism: building community capacity to effect social change. Proceedings of the SIGCHI Conference on Human Factors in Computing Systems.

[134] Madan Kumar P, Mohandoss A, Walls T, Rooban T, Vernon L (2016). Using smartphone video "selfies" to monitor change in toothbrushing behavior after a brief intervention: a pilot study. Indian J Dent Res.

[135] Almeida T, Comber R, Wood G, Saraf D, Balaam M (2016). On looking at the vagina through labella. Proceedings of the 2016 CHI Conference on Human Factors in Computing Systems.

[136] Karisalmi N, Kaipio J, Lahdenne P (2018). Improving patient experience in a children's hospital: new digital services for children and their families. Building Continents of Knowledge in Oceans of Data: The Future of Co-Created eHealth.

[137] Karisalmi N, Nieminen M (2017). Selfies of sickness: the use of video diaries with chronically ill children. Building Capacity for Health Informatics in the Future.

[138] Pollack AH, Snyder J (2021). Reflecting on patient-generated photographs of the pediatric renal transplant experience. Pediatr Transplant.

[139] Braun V, Clarke V (2006). Using thematic analysis in psychology. Qual Res Psychol.

[140] Burns K, Tuzovic S (2019). Chapter 22: Conceptualizing health consumer engagement: an extended framework of resource integration, co-creation and engagement. Handbook of Research on Customer Engagement.

[141] World Health Organization (2010). International Statistical Classification of Diseases and Related Health Problems. 10th revision.

[142] Porter M, Lee T The strategy that will fix health care. Harvard Business Review.

[143] Teisberg E, Wallace S, O’Hara S (2020). Defining and implementing value-based health care. Acad Med.

[144] Abdolkhani R, Gray K, Borda A, DeSouza R (2019). Patient-generated health data management and quality challenges in remote patient monitoring. JAMIA Open.

[145] Zimmerman M (2000). Empowerment theory. Handbook of Community Psychology.

[146] Catalani C, Minkler M (2010). Photovoice: a review of the literature in health and public health. Health Educ Behav.

[147] Wang C, Burris MA (1997). Photovoice: concept, methodology, and use for participatory needs assessment. Health Educ Behav.

[148] Harper D (2002). Talking about pictures: a case for photo elicitation. Visual Stud.

[149] Schwartz D (1989). Visual ethnography: using photography in qualitative research. Qual Sociol.

[150] boyd D (2007). Why youth (heart) social network sites: the role of networked publics in teenage social life. MacArthur Foundation Series on Digital Learning – Youth, Identity, and Digital Media Volume.

[151] Daft RL, Lengel RH (1986). Organizational information requirements, media richness and structural design. Manag Sci.

[152] Ishii K, Lyons M, Carr S (2019). Revisiting media richness theory for today and future. Human Behav Emerg Technol.

[153] Zhang Y, Parker AG (2020). Eat4Thought: a design of food journaling. Proceedings of the Extended Abstracts of the 2020 CHI Conference on Human Factors in Computing Systems.

[154] Coughlin S, Roberts D, O'Neill K, Brooks P (2018). Looking to tomorrow's healthcare today: a participatory health perspective. Intern Med J.

[155] Chakrabarti S (2014). What's in a name? Compliance, adherence and concordance in chronic psychiatric disorders. World J Psychiatry.

[156] Denecke K, Bamidis P, Bond C, Gabarron E, Househ M, Lau AY, Mayer MA, Merolli M, Hansen M (2015). Ethical issues of social media usage in healthcare. Yearb Med Inform.

[157] Chretien KC, Kind T (2013). Social media and clinical care. Circulation.

[158] Yagerman S, Marghoob A (2013). Melanoma patient self-detection: a review of efficacy of the skin self-examination and patient-directed educational efforts. Expert Rev Anticancer Ther.

[159] Lober WB, Evans HL (2019). Patient-generated health data in surgical site infection: changing clinical workflow and care delivery. Surg Infect (Larchmt).

[160] Bowen AC, Burns K, Tong SY, Andrews RM, Liddle R, O'Meara IM, Westphal DW, Carapetis JR (2014). Standardising and assessing digital images for use in clinical trials: a practical, reproducible method that blinds the assessor to treatment allocation. PLoS One.

[161] Yap MH, Chatwin KE, Ng C, Abbott CA, Bowling FL, Rajbhandari S, Boulton AJ, Reeves ND (2018). A new mobile application for standardizing diabetic foot images. J Diabetes Sci Technol.

[162] Shen D, Wu G, Suk H (2017). Deep learning in medical image analysis. Annu Rev Biomed Eng.

[163] Lundervold A, Lundervold A (2019). An overview of deep learning in medical imaging focusing on MRI. Z Med Phys.

[164] Yang Q, Steinfeld A, Zimmerman J (2019). Unremarkable AI: fitting intelligent decision support into critical, clinical decision-making processes. Proceedings of the 2019 CHI Conference on Human Factors in Computing Systems.

[165] Liu X, Faes L, Kale A, Wagner S, Fu D, Bruynseels A, Mahendiran T, Moraes G, Shamdas M, Kern C, Ledsam J, Schmid M, Balaskas K, Topol E, Bachmann L, Keane P, Denniston A (2019). A comparison of deep learning performance against health-care professionals in detecting diseases from medical imaging: a systematic review and meta-analysis. Lancet Digit Health.

[166] Dyer KA (2001). Ethical challenges of medicine and health on the internet: a review. J Med Internet Res.

[167] Palen L, Dourish P (2003). Unpacking "privacy" for a networked world. Proceedings of the SIGCHI Conference on Human Factors in Computing Systems.

[168] (2021). Health literacy-2021. Australian Medical Association.

