# Supplementary material for: Patient-Generated Health Photos and Videos Across Health and Well-being Contexts: Scoping Review
Source: J Med Internet Res. 2022 Apr 12;24(4):e28867. doi: 10.2196/28867 (PMC9044143; doi:10.2196/28867)
Supplement: Multimedia Appendix 1 [file jmir_v24i4e28867_app1.docx]

**Preferred Reporting Items for Systematic reviews and Meta-Analyses extension for Scoping Reviews (PRISMA-ScR) Checklist**

| **SECTION** | **ITEM** | **PRISMA-ScR CHECKLIST ITEM** | **REPORTED ON PAGE #** |
| --- | --- | --- | --- |
| **TITLE** | | | |
| Title | 1 | Patient-Generated Health Photos and Videos Across Health and Well-being Contexts: Scoping Review | 1 |
| **ABSTRACT** | | | |
| Structured summary | 2 | Background: Patient-generated health data are increasingly used to record health and well-being concerns and engage patients in clinical care. Patient-generated photographs and videos are accessible and meaningful to patients, making them especially relevant during the current COVID-19 pandemic to provide ongoing care. However, a systematic review of photos and videos used by patients across different areas of health and well-being is lacking.  Objectives: This review aims to synthesize the existing literature on the health and well-being contexts in which patient-generated photos and videos are used, the value gained by patients and health professionals, and the challenges experienced.  Methods: Guided by a framework for scoping reviews, we searched eight health databases (CINAHL, Cochrane Library, EMBASE, PsycINFO, PubMed, MEDLINE, Scopus, and Web of Science) and one computing database (ACM), returning a total of 28,567 studies. After removing duplicates and screening based on the predefined inclusion criteria, we identified 110 relevant articles. Data were charted and articles were analyzed following an iterative thematic approach with the assistance of NVivo software (version 12; QSR International).  Results: Patient-generated photos and videos are used across a wide range of health care services (39/110, 35.5% articles), for example, to diagnose skin lesions, assess dietary intake, and reflect on personal experiences during therapy. In addition, patients use them to self-manage health and well-being concerns (33/110, 30%) and to share personal health experiences via social media (36/110, 32.7%). Photos and videos create significant value for health care (59/110, 53.6%), where images support diagnosis, explanation, and treatment (functional value). They also provide value directly to patients through enhanced self-determination (39/110, 35.4%), social (33/110, 30%), and emotional support (21/110, 19.1%). However, several challenges emerge when patients create, share, and examine photos and videos, such as limited accessibility (16/110, 14.5%), incomplete image sets (23/110, 20.9%), and misinformation through photos and videos shared on social media (17/110, 15.5%).  Conclusions: This review shows that photos and videos engage patients in meaningful ways across different health care activities (eg, diagnosis, treatment, and self-care) for various health conditions. Although photos and videos require effort to capture and involve challenges when patients want to use them in health care, they also engage and empower patients, generating unique value. This review highlights areas for future research and strategies for addressing these challenges.  Keywords: patient engagement; patient-generated health data; consumer-generated health data; personal health information; patient empowerment; mobile phone | 2 |
| **INTRODUCTION** | | | |
| Rationale | 3 | A rationale for the paper is that photographs and videos are meaningful to patients who are already using them to track their health, they are of increasing use during COVID-19 and a comprehensive assessment of the extent of research evidence and potential scope of patient-generated photos and videos in different areas of health and wellbeing seems to be lacking. | 3-4 |
| Objectives | 4 | The overarching objective of this review is to synthesize the literature on the uses, values and challenges with patient-generated photos and videos across health and wellbeing contexts. Specific objectives include (1) providing an overview of the contexts in which photos and videos are used, (2) examining the value gained for patients, caregivers, healthcare professionals, and other stakeholders, (3) as well as the challenges experienced by these groups in creating, sharing, and examining photos and videos. | 4 |
| **METHODS** | | | |
| Protocol and registration | 5 | A scoping review protocol was not published. | 4 |
| Eligibility criteria | 6 | The search included articles from January 2008 to 31 January 2021 in English language. The start date was chosen because the major brands of smartphones - iPhone and Android – were first released in 2007 and 2008, respectively, which provide the platform for patient-generated photos and videos. Articles in other languages were excluded because of the cost and time required in translating. Only peer-reviewed articles which included primary research were selected to ensure conclusions were supported by an evidence base. According to the objectives of this study and the focus on patients as technology users, we conducted our search strategy in both health and computing databases.  Inclusion criteria  1. Articles describe patient-generated photography or videos that reflect personal information and experiences to help address a health and wellbeing concern  2. Photos or videos are taken by patients, carers or other participants that are not healthcare professionals or researchers  3. Findings report on photography or video as a collection mechanism, intervention, or unit of analysis  Exclusion criteria  1. Publications without primary research, such as editorials, opinions, perspectives, reviews, research protocols  2. Secondary analysis of photos and videos, e.g., from social media, that have been shared by individuals without an explicit health or wellbeing intent  3. Automatic video recordings of consultations or teleconsultations as well as images generated by clinician, surveillance and patient monitoring systems | 5-7 |
| Information sources* | 7 | According to the objectives of this study and the focus on patients as technology users, we conducted our search strategy in both health and computing databases. Further, we considered social science databases such as EMBASE and PsycINFO to cover special studies in psychology and behavioural science. We searched through 8 health databases (Cumulative Index to Nursing and Allied Health Literature, Cochrane Library, EMBASE, PsycINFO, Web of Science, PubMed, MEDLINE, and Scopus) and 1 computing database (ACM). To ensure that we did not neglect any relevant article, we broadened the search using MeSH terms and synonyms to collect a comprehensive pool of relevant articles. As illustrated in Figure 1, the health database search yielded 28,026 results and the ACM search yielded 541 results. Besides, two authors (BP, KB) hand-searched the reference lists of related review articles and JMIR archives, which returned 17 additional articles. | 6-7 |
| Search | 8 | image* OR pictur* OR photo* OR video* OR selfie* OR portrait* OR snap* OR shot* OR depict* OR data* OR info*)  AND  (patient* OR consumer* OR care* OR customer* OR veteran* OR client* OR self* OR crowd*)  AND  (generate* OR record* OR creat* OR captur* OR document* OR evidence* OR story OR report* OR track* OR initiat* OR monitor* OR take*). | 5 |
| Selection of sources of evidence† | 9 | To answer our research questions, we collated the results of the thematic analysis identifying key themes in the 110 articles related to the use photos and videos, the value gained, and the challenges experienced. A selection of 15 articles was coded independently by all three authors using the chosen frameworks. Regular meetings were health to explore any discrepancies and the suitability of the frameworks for our objectives. Once agreement had been reached one author (BP) coded the remaining papers. Next, through discussion with all authors, we structured the codes into 3 themes based on our objectives of understanding (1) contexts, (2) values, and (3) challenges, as well as sub-themes to generate knowledge and identify gaps. | 7-10 |
| Data charting process‡ | 10 | The data was charted by two authors in a pre-defined form that collated publication data and information relevant to our research questions (e.g., author information, year published, aims, target group, intervention, research methods, results, conclusions, gaps, uses, value and challenges). However, to manage the large volume of data generated through charting, NVivo 12 was chosen as an alternative, and the articles were coded for the elements in the pre-defined form. Publication data was extracted verbatim from each article by two authors, whereas coding and critical analysis for the research questions was completed by all authors. Extracted information was discussed at regular meetings of all authors to ensure the research questions were still relevant, the articles could answer the research questions and to explore any to clarify key concepts and identify major gaps. | 7-10 |
| Data items | 11 | Information charted included author information, year published, aims, target group, intervention, research methods, number of images taken, type of technology used, who the images were generated for results, conclusions, gaps and qualitative thematic analysis for uses, value and challenges. | Multimedia Appendix 3 |
| Critical appraisal of individual sources of evidence§ | 12 | Articles included in this review comprise diverse study designs and target cohorts, and outcomes. A formal assessment of study quality was not undertaken because this was a scoping review where most studies published have been pilot or feasibility studies. The review included no randomised control trials, which is not surprising since photos and videos are often patient driven. | 23 |
| Synthesis of results | 13 | Following the recommendation of Arksey and O’Malley [20], we collated and reported the results based on a thematic analysis approach [139] with an analytic framework [140]. Our thematic analysis followed the steps described by Braun, Clarke [139]. We started by reading articles to familiarize ourselves with the data, recorded notes through “memo” and “annotation” features of NVivo and discussed ideas for coding. One author (AR) manually coded a subset of the 110 articles to generate an initial list of 102 codes relevant to our research questions of health and wellbeing contexts, value generated, and challenges. These initial codes gave us an overview of the data, but it also highlighted the diversity of study designs and results, which made the aggregation of findings impossible. Instead, we needed a framework to structure and report the results according to our research questions.  To structure the results around health contexts, we initially coded articles according to the International Classification of Diseases, 10th revision (ICD-10) [141], a medical classification established by the World Health Organization (WHO) consisting of 21 chapters. However, we found this framework limited, because it presented a medical perspective and did not fit well to articles that reported wellbeing outcomes or social media contexts. Hence, we revised the structure around the primary contexts presented in the articles: (1) healthcare services, where patients share images with a healthcare professional to observe and treat health and wellbeing concerns; (2) self-management, where patients use images to independently track and manage health concerns; (3) social media, where patients share personal health information and experiences with peers online; (4) education, where images are used for health education in schools and waiting rooms; and (5) service improvement, where patients are invited to take images to reflect on their health service experience and to express their needs.  To analyse the value of photos and videos reported in our article collection, we employed a health consumer engagement framework [140] which highlights six key values of PGHD: functional, emotional, social, transactional, efficiency, and self-determination value.  To analyse the challenges, we identified several frameworks that describe data challenges [27,144]. Table 3 presents the full list of challenges identified and how we operationalized them for our analysis. | 8-9 |
| **RESULTS** | | | |
| Selection of sources of evidence | 14 | A PRISMA flow diagram is reported in the paper to show sources of evidence screened, assessment for eligibility and exclusions. | 7 |
| Characteristics of sources of evidence | 15 | A summary table of papers has been provided in Multimedia Appendix 3 | Multimedia Appendix 3 |
| Critical appraisal within sources of evidence | 16 | A summary table of papers has been provided in Multimedia Appendix 3 | Multimedia Appendix 3 |
| Results of individual sources of evidence | 17 | A summary table of papers has been provided in Multimedia Appendix 3 | Multimedia Appendix 3 |
| Synthesis of results | 18 | Of the 110 articles identified in this review, 90 (81.8%) reported on photos and 23 (20.9%) used videos (3 of these articles employed both photos and videos). Figure 2 provides an overview of the key themes revealed in our review, showing the contexts in which photos and videos are used, values gained by patients, and challenges when taking, sharing and examining photos and videos.  As summarized in Table 1, images were largely used in healthcare service, self-management, and social media contexts. Multimedia Appendix 3 provides a more detailed table that also lists who captured the images (patient or carer), the technologies used to capture and share images, and the audiences receiving them.  Patient-generated photos and videos create significant value when used for health and wellbeing. Based on an engagement framework [140], our analysis identified six key values: functional, self-determination, social, emotional, transactional, and efficiency value. Table 2 provides a summary of each value and the number of relevant articles.  The final part of our analysis describes the barriers and challenges faced by patients with health health-related photographs and videos. Here, our analysis is structured based on the process of working with photos and videos, starting with challenges that patients face when they take photos, share them with peers and health professionals, and when they are examined. These challenges are interrelated, meaning that challenges in taking photos and sharing them, in turn, can also affect examination. Table 3 provides a summary | 10, 11, 14, 17-18 |
| **DISCUSSION** | | | |
| Summary of evidence | 19 | Patient-generated photos and videos are used across a wide range of healthcare services (39/110 articles, 35.4%), e.g., to diagnose skin lesions, assess dietary intake, and reflect on personal experiences during therapy. Beyond that, patients use them to self-manage health and wellbeing concerns (33/110, 30%) and to share personal health experiences via social media (36/110, 32.7%). Photos and videos create significant value for healthcare (59/110, 53.6%), where images support diagnosis, explanation, and treatment (functional value). Photos also provide value directly to patients through enhanced self-determination (39/110, 35.4%), social (33/110, 30.0%) and emotional support (21/110, 19.1%). However, several challenges emerge when patients create, share, and examine photos and videos, such as limited accessibility (16/110, 14.5%), incomplete photo sets (23/110, 20.9%), and misinformation through photos and videos shared on social media (17/110, 15.4%). | 21-23 |
| Limitations | 20 | Our review is subject to several limitations. First, our inclusion criteria limited our review results to only English-language articles and published peer review literature from 2008 to January 2021. Second, the articles included in this review comprise diverse study designs and target cohorts, and outcomes. A formal assessment of study quality was not undertaken because this was a scoping review, where the majority of studies published have been pilot or feasibility studies. Third, synthesising outcomes from a large collection of diverse studies across different contexts was challenging. Only a subset of papers reported health outcomes (reported under functional value). Many papers presented formative research on the feasibility of introducing photos and videos into a particular context, or on the experiences and motivations of patients and healthcare professionals. Hence, instead of outcomes, we framed the results sections more broadly around the various contexts, the value generated for patients and healthcare professionals, and their challenges. The value for patients and healthcare professionals was analysed and collated based on an established framework on the value of PGHD. The analysis of contexts and challenges was largely inductive, because existing frameworks for PGHD did not cover the specifics of photos and videos such as challenges with the photo quality or emotional labour. To ensure consistency, the analysis was conducted independently by three members of the research team. Finally, the broad scope of this review and the large number of articles did not allow for a comparison of effects. | 23-24 |
| Conclusions | 21 | This review shows that photos and videos are engaging patients in meaningful ways across different health and wellbeing contexts. While photos and videos require effort in capture and involve challenges when patients want to use them in healthcare, they are also engaging and empowering patients, generating unique value. This review highlights areas for future research and strategies to address identified challenges. | 27-28 |
| **FUNDING** | | | |
| Funding | 22 | This review has been supported by Queensland University of Technology (QUT) and The Prince Charles Hospital Foundation (Grant # INN2018-36). | 28 |

JBI = Joanna Briggs Institute; PRISMA-ScR = Preferred Reporting Items for Systematic reviews and Meta-Analyses extension for Scoping Reviews.

* Where *sources of evidence* (see second footnote) are compiled from, such as bibliographic databases, social media platforms, and Web sites.

† A more inclusive/heterogeneous term used to account for the different types of evidence or data sources (e.g., quantitative and/or qualitative research, expert opinion, and policy documents) that may be eligible in a scoping review as opposed to only studies. This is not to be confused with *information sources* (see first footnote).

‡ The frameworks by Arksey and O’Malley (6) and Levac and colleagues (7) and the JBI guidance (4, 5) refer to the process of data extraction in a scoping review as data charting*.*

§ The process of systematically examining research evidence to assess its validity, results, and relevance before using it to inform a decision. This term is used for items 12 and 19 instead of "risk of bias" (which is more applicable to systematic reviews of interventions) to include and acknowledge the various sources of evidence that may be used in a scoping review (e.g., quantitative and/or qualitative research, expert opinion, and policy document).

*From:* Tricco AC, Lillie E, Zarin W, O'Brien KK, Colquhoun H, Levac D, et al. PRISMA Extension for Scoping Reviews (PRISMAScR): Checklist and Explanation. Ann Intern Med. 2018;169:467–473. [doi: 10.7326/M18-0850](http://annals.org/aim/fullarticle/2700389/prisma-extension-scoping-reviews-prisma-scr-checklist-explanation).
