# Supplementary material for: Patient-Generated Health Photos and Videos Across Health and Well-being Contexts: Scoping Review
Source: J Med Internet Res. 2022 Apr 12;24(4):e28867. doi: 10.2196/28867 (PMC9044143; doi:10.2196/28867)
Supplement: Multimedia Appendix 2 [file jmir_v24i4e28867_app2.docx]

| Database | Strategy 2008 - Jan 31, 2021 |
| --- | --- |
| **CINAHL Complete (via EBSCOhost)**  Results: **3258** | (image* OR pictur* OR photo* OR video* OR selfie* OR portrait* OR snap* OR shot* OR depict* OR data* OR info*) **TI**  ***AND***  (patient* OR consumer* OR care* OR customer* OR veteran* OR client* OR self* OR crowd*) **TI**  ***AND***  (generate* OR record* OR creat* OR captur* OR document* OR evidence* OR story OR report* OR track* OR initiat* OR monitor* OR take*) **TI**  Peer-reviewed journal article Jan 2008-Jan 2021 in English |
| **Cochrane**    Results:**5** | (image* OR pictur* OR photo* OR video* OR selfie* OR portrait* OR snap* OR shot* OR depict* OR data* OR info*) **TI**  ***AND***  (patient* OR consumer* OR care* OR customer* OR veteran* OR client* OR self* OR crowd*) **TI**  ***AND***  (generate* OR record* OR creat* OR captur* OR document* OR evidence* OR story OR report* OR track* OR initiat* OR monitor* OR take*) **TI**  Peer-reviewed journal article 2008-2021 in English (Cochrane Reviews) |
| **Embase**    Results:**7,273** | (image* OR pictur* OR photo* OR video* OR selfie* OR portrait* OR snap* OR shot* OR depict* OR data* OR info*) **TI**  ***AND***  (patient* OR consumer* OR care* OR customer* OR veteran* OR client* OR self* OR crowd*) **TI**  ***AND***  (generate* OR record* OR creat* OR captur* OR document* OR evidence* OR story OR report* OR track* OR initiat* OR monitor* OR take*) **TI**  Peer-reviewed journal articles 2008-2021 in English |

| **Medline  (via Ebscohost)**    Results:**3,371** | (image* OR pictur* OR photo* OR video* OR selfie* OR portrait* OR snap* OR shot* OR depict* OR data* OR info*) **TI**  ***AND***  (patient* OR consumer* OR care* OR customer* OR veteran* OR client* OR self* OR crowd*) **TI**  ***AND***  (generate* OR record* OR creat* OR captur* OR document* OR evidence* OR story OR report* OR track* OR initiat* OR monitor* OR take*) **TI**  Peer-reviewed journal article 2008-2021 in English (+Human) |
| --- | --- |
| **PsycInfo (via Ovid)**    Results: **1014** | (image* OR pictur* OR photo* OR video* OR selfie* OR portrait* OR snap* OR shot* OR depict* OR data* OR info*) **TI**  ***AND***  (patient* OR consumer* OR care* OR customer* OR veteran* OR client* OR self* OR crowd*) **TI**  ***AND***  (generate* OR record* OR creat* OR captur* OR document* OR evidence* OR story OR report* OR track* OR initiat* OR monitor* OR take*) **TI**  Peer-reviewed journal article 2008-2021 in English |
| **Pubmed**  Results:**3,374** | (image* OR pictur* OR photo* OR video* OR selfie* OR portrait* OR snap* OR shot* OR depict* OR data* OR info*) **TI**  ***AND***  (patient* OR consumer* OR care* OR customer* OR veteran* OR client* OR self* OR crowd*) **TI**  ***AND***  (generate* OR record* OR creat* OR captur* OR document* OR evidence* OR story OR report* OR track* OR initiat* OR monitor* OR take*) **TI**  Peer-reviewed journal article 2008-2021 in English (+Human) |

| **Scopus**    Results: **6,365** | (image* OR pictur* OR photo* OR video* OR selfie* OR portrait* OR snap* OR shot* OR depict* OR data* OR info*) **TI**  ***AND***  (patient* OR consumer* OR care* OR customer* OR veteran* OR client* OR self* OR crowd*) **TI**  ***AND***  (generate* OR record* OR creat* OR captur* OR document* OR evidence* OR story OR report* OR track* OR initiat* OR monitor* OR take*) **TI**  Peer-reviewed journal article 2008-2021 in English |
| --- | --- |
| **Web of Science**  Results:**3366** | (image* OR pictur* OR photo* OR video* OR selfie* OR portrait* OR snap* OR shot* OR depict* OR data* OR info*) **TI**  ***AND***  (patient* OR consumer* OR care* OR customer* OR veteran* OR client* OR self* OR crowd*) **TI**  ***AND***  (generate* OR record* OR creat* OR captur* OR document* OR evidence* OR story OR report* OR track* OR initiat* OR monitor* OR take*) **TI**  Peer-reviewed journal articles + reviews 2008-2021 in English (Science + Social Science databases) |
| **The ACM Guide to Computing Literature**  Results: **541** | *Title:(image* OR pictur* OR photo* OR video* OR selfie* OR portrait* OR snap* OR shot* OR depict* OR data* OR info*)*  **AND**  *Title:(:(patient* OR consumer* OR care* OR customer* OR veteran* OR client* OR self* OR crowd* OR phone*))*  **AND**  *Title:((generate* OR record* OR creat* OR captur* OR document* OR evidence* OR story OR report* OR track* OR initiat* OR monitor* OR take*) )*  Peer-reviewed research articles 2008-2021 in English |
| **28,026** | HEALTH TOTAL |
| **541** | ACM TOTAL RESULTS |
| **18,550** | DUPLICATES REMOVED |
| **10,017** | TOTAL RESULTS AFTER DUPLICATES REMOVED |
