# Supplementary material for: Patient-Generated Health Photos and Videos Across Health and Well-being Contexts: Scoping Review
Source: J Med Internet Res. 2022 Apr 12;24(4):e28867. doi: 10.2196/28867 (PMC9044143; doi:10.2196/28867)
Supplement: Multimedia Appendix 4 [file jmir_v24i4e28867_app4.docx]

Appendix 4 Coding Tree

## Exported from NVivo 12 on 21 Dec 2021.

## Notes:

## Files refers to the number of articles that contain a code; references refers to the total number of times a code has been applied to show when codes appear multiple times in an article.

## The coding tree includes all codes generated, including codes that are not presented in the paper (such as the health areas specified by ICD-10 chapters) as well as more specific sub-codes for contexts and challenges identified.

| Name | Files | References |
| --- | --- | --- |
| **Miscellaneous** | **10** | **14** |
| Why photo | 8 | 11 |
| Why video | 2 | 3 |
| **RQ1 Health Areas** | **110** | **213** |
| ICD_10_CM chapters | 110 | 209 |
| 1- Certain infectious and parasitic diseases | 6 | 6 |
| B95 - B98 Bacteria, viral and other infections agents | 4 | 4 |
| 10- Diseases of the respiratory system | 0 | 0 |
| 11- Diseases of the digestive system | 5 | 6 |
| K40 - K46 Hernia | 2 | 2 |
| 12- Diseases of the skin and subcutaneous tissue | 4 | 8 |
| L00 - L08 Infections of the skin and subcutaneous tissue | 4 | 4 |
| L40 - L 45 Papulosquamous disorders | 2 | 3 |
| 13- Diseases of the musculoskeletal system and connective tissue | 0 | 0 |
| 14- Diseases of the genitourinary system | 4 | 7 |
| N20 - N23 Urolithiasis | 1 | 2 |
| N60 - N64 Disorders od breast | 1 | 3 |
| 15- Pregnancy, childbirth and the puerperium | 0 | 0 |
| 16-Certain conditions originating in the perinatal period | 0 | 0 |
| 17- Congenital malformations, deformations and chromosomal abnormalities | 0 | 0 |
| 18- Symptoms, signs and abnormal clinical and laboratory findings, not elsewhere classified | 1 | 1 |
| 19- Injury, poisoning and certain other consequences of external causes | 7 | 13 |
| S30 - S39 Injuries to the abdomen, lower back, lumbar spine and pelvis | 0 | 0 |
| T00 - T07 Injuries involving multiple body regions | 1 | 1 |
| T81 - Complications of procedures, not elsewhere classified | 4 | 4 |
| T81.4 Infections of procedures, notelsewhere classified | 2 | 2 |
| 2- Neoplasms | 13 | 30 |
| C49 - Maligant neoplasm of other connective and soft tissue | 2 | 2 |
| Cancer | 4 | 5 |
| Breast Cancer | 1 | 2 |
| Skin Cancer | 4 | 16 |
| 20- External causes of morbidity | 1 | 1 |
| X60 - X84 Intentional self-harm | 1 | 1 |
| 21- Factors influencing health status and contact with health services | 7 | 7 |
| Z70 - Z76 Persons encountering health services in other circumstances | 5 | 5 |
| 3- Diseases of the blood and blood-forming organs and certain disorders involving the immune mechanism | 0 | 0 |
| 4- Endocrine, nutritional and metabolic diseases | 42 | 85 |
| Nutrition | 32 | 69 |
| E50 - E64 other nutritional deficiencies | 1 | 4 |
| E65 - E68 Obesity and other hyperalimentation | 7 | 10 |
| Improve reporting accuracy | 5 | 7 |
| T2DM | 4 | 10 |
| 5- Mental, Behavioral and Neurodevelopmental disorders | 19 | 38 |
| F17.218 Nicotine dependence, cigarettes, with other nicotine-induced disorders | 6 | 15 |
| F30 - F39 Mood [affective] disorders | 2 | 2 |
| F99 - F99 Unspecified mental disorders | 2 | 2 |
| R63 Symptoms and signs concerning food and fluid intake | 4 | 8 |
| 6- Diseases of the nervous system | 2 | 2 |
| G30 - G32 other degenerative diseases of other nervous system | 1 | 1 |
| 7- Diseases of the eye and adnexa | 0 | 0 |
| 8- Diseases of the ear and mastoid process | 0 | 0 |
| 9- Diseases of the circulatory system | 3 | 3 |
| 20 - I25 ischaemic heart diseases | 1 | 1 |
| **RQ1 Health Contexts** | **110** | **309** |
| C Education | 7 | 19 |
| C Education (in school) | 3 | 10 |
| C Education (outside school, eg waiting room, home) | 4 | 9 |
| C Healthcare Service | 39 | 105 |
| C Clinical - Diagnostic aid | 6 | 16 |
| C Clinical - dietary assessment with healthcare professional (endocrinologist, dietitian) | 12 | 31 |
| C Clinical - Other | 3 | 9 |
| C Clinical - Shared in consultation | 2 | 9 |
| C Clinical - Shared in follow up (treatment) | 13 | 29 |
| C Clinical - Shared therapy | 3 | 11 |
| C Self Management | 33 | 80 |
| C Self-management - dietary self monitoring | 23 | 55 |
| C Self-management - mental health and wellbeing | 5 | 17 |
| C Self-management - smoking cessation | 4 | 6 |
| C Service Improvement (similar to photo or video elicitation study) | 3 | 11 |
| C Social Media | 36 | 94 |
| C Social Media other | 0 | 0 |
| C Social media photos and captions to disclose health information | 19 | 50 |
| C Social Media videos to share health experience | 17 | 44 |
| **RQ2 Value creation** | **109** | **366** |
| Efficiency value | 19 | 27 |
| Emotional value | 21 | 48 |
| Functional value | 59 | 101 |
| Self-determination value | 39 | 68 |
| Social value | 33 | 81 |
| Transactional value | 18 | 40 |
| **RQ3 Barriers and challenges experienced by customer and HCP - selfies as a process or practice** | **76** | **201** |
| 3A Creation challenges - invisible labour (medical work by patients) | 40 | 85 |
| 3Aa Accessibility and usability barriers in taking photos | 16 | 21 |
| 3 difficult to reach and take photo of body part (may also be in accessibility barriers) | 2 | 2 |
| 3b social challenge - not appropriate to take health photo in public or a taboo | 7 | 7 |
| 3Ab Adoption patients (use over time) - time and effort required to take up and maintain engagement over longer periods of time | 7 | 10 |
| 3Ac Incomplete data (forget, not enough time, too much effort required) 3 Consistency - incomplete data, lapses; includes also sub-nodes | 23 | 35 |
| 3 contextual data missing (limits interpretability) | 5 | 6 |
| 3 life disruption | 2 | 2 |
| 3B lapse data collection when goals change | 1 | 1 |
| 3Ad Accuracy or photo quality (enough detail, in focus, lighting ...) | 16 | 19 |
| 3B Sharing challenges of data with health professionals and peers | 33 | 71 |
| 3B1 health professionals adoption - time and effort required to prompt and check data | 4 | 5 |
| 3 Responsibility between patient and health professional not clearly shared, ie if data is shared to indicate potential health concern | 1 | 1 |
| 3B1 Integration (institutional environment and IT or EHR infrastructure) | 1 | 1 |
| 3B2a PRIVACY patient privacy a concern when data shared with others, both health professionals and peers online (boyd - persistence, searchability, exact copyability, and invisible audiences, also concern about lack of secure transfer and storage) | 10 | 15 |
| 3B2b PRIVACY health professionals privacy of health professionals and their families | 1 | 1 |
| 3B3 Social Media MISINFORMATION or promotion of unhealthy or harmful behaviours on social media that share PGHD | 17 | 41 |
| 3 opposite of social value - photo interventions may have opposite effect of entrenching people in harmful behaviours | 2 | 3 |
| 3B4 social media - inappropriate feedback (rude, harmful, victim blaming, spam) | 7 | 9 |
| 3C Examination challenges | 21 | 42 |
| 3C1 Interpretability (mis-diagnosis, misinterpretation, getting nutrition value in photos wrong, giving false reassurance when there might be health issue) | 10 | 15 |
| 3 potential mis-diagnosis or misinterpretation | 2 | 3 |
| 3C2 Relevancy and timeliness | 6 | 11 |
| 3 lack of representation of diverse patients in photos | 1 | 1 |
| 3 Timeliness | 2 | 3 |
| 3C3 Emotional Labour - revisit past photos, new diagnosis, anxiety about health concern when there is none | 7 | 16 |
| 3 anxiety or concern that there may be a health concern when there is none | 1 | 1 |
| 3 Emotional labour of revisiting past photos which can trigger negative emotions or emotional labour of diagnosing a new health problem | 6 | 7 |
